# Psychosocial and Demographic Factors Associated with Physical Multimorbidity in Severe Mental Illness: A Systematic Review

**DOI:** 10.1093/schbul/sbaf128

**Published:** 2025-08-28

**Authors:** Erin G Lawrence, Uzma Zahid, Abigail C Thomson, Robin Lau, Laura Havers, Federica Biotti, Monica Acosta Pereira, Mark C Freestone, Irene Gonzalez-Calvo, Brent Elliott, Kamaldeep Bhui, Georgina M Hosang

**Affiliations:** Centre for Psychiatry and Mental Health, Wolfson Institute of Population Health, Queen Mary University of London, London, United Kingdom, E1 2AB; Department of Psychology, Institute of Psychiatry, Psychology and Neuroscience, Kings College London, London, United Kingdom, SE5 8AB; Centre for Psychiatry and Mental Health, Wolfson Institute of Population Health, Queen Mary University of London, London, United Kingdom, E1 2AB; Division of Psychology and Language Sciences, University College London, London, United Kingdom; Camden and Islington NHS Foundation Trust, London, United Kingdom, NW1 0PE; Centre for Psychiatry and Mental Health, Wolfson Institute of Population Health, Queen Mary University of London, London, United Kingdom, E1 2AB; Centre for Psychiatry and Mental Health, Wolfson Institute of Population Health, Queen Mary University of London, London, United Kingdom, E1 2AB; Centre for Psychiatry and Mental Health, Wolfson Institute of Population Health, Queen Mary University of London, London, United Kingdom, E1 2AB; Centre for Psychiatry and Mental Health, Wolfson Institute of Population Health, Queen Mary University of London, London, United Kingdom, E1 2AB; The Tavistock and Portman NHS Foundation Trust, London, United Kingdom, NW3 5BA; Centre for Psychiatry and Mental Health, Wolfson Institute of Population Health, Queen Mary University of London, London, United Kingdom, E1 2AB; East London NHS Foundation Trust, London, United Kingdom, E1 8DE; Centre for Psychiatry and Mental Health, Wolfson Institute of Population Health, Queen Mary University of London, London, United Kingdom, E1 2AB; East London NHS Foundation Trust, London, United Kingdom, E1 8DE; Department of Psychiatry, and Wadham College, University of Oxford, Oxford, United Kingdom, OX3 7JX; NIHR Oxford Health Biomedical Research Centre and Oxford Health NHS Trust, Oxford, United Kingdom, OX3 7JX; Global Policy Institute, Queen Mary University of London, London, United Kingdom; Collaborating Centre of World Psychiatric Association, Oxford, United Kingdom; Centre for Psychiatry and Mental Health, Wolfson Institute of Population Health, Queen Mary University of London, London, United Kingdom, E1 2AB

**Keywords:** psychosis, premature mortality, physical comorbidities, schizophrenia, bipolar disorder, psychosocial risk factors, demographic risk factors

## Abstract

**Background and Hypothesis:**

People with severe mental illness (SMI), such as schizophrenia and bipolar disorder have a reduced life expectancy. This is largely due to physical multimorbidity (MM), defined as the coexistence of two or more physical health conditions. This systematic review identifies which psychosocial and demographic factors are associated with MM in SMI.

**Study Design:**

Embase, PubMed, and PsychINFO were searched with no limits on publication date or study design. Studies were eligible for inclusion if they assessed the impact of psychosocial and/or demographic factors on MM outcomes among people with SMI.

**Study Results:**

Thirty studies met the inclusion criteria for this review. The strongest predictors of MM were childhood maltreatment (odds ratios [OR] up to 8.70 [95% CI 2.49-30.33]), female gender (OR up to 2.47 [95% CI 1.35-4.50]), older age (OR up to 1.60 [95% CI 1.31-1.96]), and ethnicity (e.g. OR up to 2.09 [95% CI 1.81-2.42] for Black Caribbean groups relative to White British groups). Predictors with mixed evidence included educational attainment, employment status, socioeconomic status, marital status, urbanicity, deprivation, country of origin, healthcare access, and global functioning.

**Conclusions:**

The findings highlight psychosocial factors (e.g. childhood maltreatment) and demographic factors (e.g. older age) that may contribute to MM, which has strong clinical implications. Some factors are modifiable (e.g. education) and can inform risk prevention strategies for MM in SMI, mitigating risks of premature mortality. Future research should use consistent definitions of MM for cross-study comparisons and assess additional risk factors, their interactions, and underlying mechanisms.

## Introduction

People with severe mental illness (SMI; such as schizophrenia, bipolar disorder, and other psychoses) have a reduced life expectancy of up to 20 years relative to the general population. ^1^^,^^2^ Premature mortality in this population is largely attributed to coexisting long-term physical illnesses,^2^^,^^3^ known as physical multimorbidity (MM). MM is commonly defined as the coexistence of two or more chronic physical health conditions (e.g. cardiovascular, respiratory, and metabolic diseases).^3^ MM can also include cancer^4^ and associated comorbidities resulting from immunosuppression.^5^^,^^6^ A recent meta-analysis found that people with SMI are 2.4 times more likely to experience MM than those without SMI.^4^ MM in SMI is associated with greater hospital readmissions,^7^^,^^8^ disability,^7^^,^^9^ increased healthcare costs,^10^^,^^11^ and can impede treatment adherence and response.^10^

Research consistently demonstrates the important role of pharmacological (e.g. antipsychotic medication), biological (e.g. pathophysiological, genetic), and lifestyle factors (e.g. smoking) in MM among people with SMI.^12–14^ However, psychosocial and demographic factors have been largely neglected in this area, even though some are modifiable and plausible intervention targets (e.g. social isolation). A recent systematic review exploring the prevalence and risk of MM in psychosis highlighted several sociodemographic factors (e.g. female sex) reported by included studies, although these were not systematically searched for.^13^ Questions remain surrounding the importance of psychosocial and demographic factors in MM in SMI warranting a systematic synthesis of the available literature.

Psychosocial factors encompass psychological attributes (e.g. distress) and social experiences (e.g. social support)^15^^,^^16^ which can act synergistically.^16^ Individuals with SMI often experience psychosocial hardship (e.g. social isolation),^17^^,^^18^ and SMI incidence is greater in minority ethnic groups, urban settings, and in lower socioeconomic groups facing poverty and adversity.^19^ Psychosocial factors (e.g. poor social support and financial challenges) are associated with cardiovascular, metabolic, and respiratory diseases in the general population.^20^ Exposure to such psychosocial stress can trigger altered biological functioning (e.g. allostatic load and inflammation), leading to disease burden over time.^21–23^

Syndemic theory suggests that psychosocial inequalities can cause co-occurring epidemics through disease interactions with psychosocial factors, leading to poorer health outcomes.^24–26^ Therefore, living with SMI while experiencing psychosocial hardship may elevate one’s risk of physical illness and MM. Thus, potentially magnifying the impact of psychosocial factors for MM among people with SMI, providing a possible explanation for the increased risk of MM in this group relative to the general population. ^4^

The aim of this study is to systematically review and synthesise the literature concerned with the association between psychosocial and demographic factors with MM in SMI.

## Methods

This systematic review was registered on PROSPERO (CRD42022382661) and conducted following the Preferred Reporting Items for Systematic Reviews and Meta-Analysis (PRISMA) guidelines^27^ (Table S9).

### Search Strategy

The review followed a PEO (Population, Exposure, Outcome) framework,^28^ whereby people with SMI were the population of interest, psychosocial and demographic risk factors were the exposure, and MM was the outcome (Table S1). Embase, PubMed, and PsychINFO were searched between March 2023 and April 2025 to retrieve publications for inclusion in this review. Search terms were applied to the title and abstract fields for: SMI (Population), risk factors (Exposure), and physical MM (Outcome). Terms for MM and psychosis were adapted from a previous systematic review.^13^ There were no limits on publication date, but only those published in English were considered.

To reduce publication bias, OATD was searched for grey literature, and the first 200 relevant publications from Google Scholar were retrieved to optimise the capture of relevant research.^29^ Articles included in previous meta-analyses on MM in SMI^4^^,^^13^ were retrieved to ensure relevant studies were not overlooked. Forward and backwards citation searches of the final studies eligible for inclusion were conducted. The full search strategy is available in Table S8. References were exported to Rayyan,^30^ and duplicates were removed before screening.

### Study Selection

Studies were eligible for inclusion if they investigated demographic or psychosocial risk factors for MM in SMI samples. This includes schizophrenia, first-episode psychosis, and other psychotic disorders listed in the ICD-10 (e.g. F20-F29), and bipolar disorder. Studies concerned with individuals with psychotic features were also considered. Studies had to investigate MM as an outcome variable for inclusion. While definitions of MM vary across research,^13^ for this review, we defined MM as having two or more chronic physical illnesses (in addition to SMI). Since there is limited research on this topic, studies that focused on the total number of physical comorbidities in addition to SMI were considered to ensure that relevant studies were not overlooked. There were no limitations on study setting or design. Review articles, abstracts, editorials, books, and opinion pieces were excluded. See Table S1 for this review’s full eligibility criteria.

EGL screened abstracts and titles of identified studies based on eligibility criteria. Second reviewers (AT, MA) independently screened a random 10% of the studies. There was strong agreement between reviewers (99.3%). Disagreements were resolved through discussion (*n* = 4) aligning with the primary reviewer’s decision (EGL). Studies meeting eligibility at this stage proceeded to full-text screening by EGL.

### Data Extraction

EGL extracted the data from included studies into a structured Microsoft Excel coding form. This was independently checked by two reviewers (UZ, RL) and unresolved discrepancies were discussed with the senior author (GH). The following details were extracted where available in the paper: author(s), publication year, country of study, study design, study MM definition, sample size, gender/sex of cases, mean age of cases, ethnicity of cases, SMI diagnoses, diagnostic methods, patient setting, presence of psychiatric comorbidities, medication use, physical illnesses studied, risk factors studied, and those associated with MM (with available effect sizes). For studies that did not use inferential statistics but reported relevant data, chi-square tests or *t*-tests (for associations, and mean levels, respectively) were conducted in R for MacOS (version 4.2.2)^31^ using the epitools (version 0.5-10.1)^32^ and BSDA (version 1.2.2)^33^ R packages. To facilitate data synthesis,^34^ Hedges’ *g* and odds ratios (OR) were calculated to estimate the effect size (for mean differences or associations, respectively) in papers that did not report these values. If data were not reported authors were contacted for further information.

### Study Quality

The methodological quality and risk of bias of the studies selected for inclusion were assessed by three authors (EGL, UZ, RL) using Joanna Briggs Institute (JBI) critical appraisal tools.^35^^,^^36^ JBI provides critical appraisal checklists appropriate for various study designs. The tools scored ‘1’ if a criterion was met and ‘0’ if it was not satisfied or omitted. The scores were summed to create a final quality rating of ‘low’, ‘moderate’, or ‘high’ quality (Table S6). Any discrepancies in the quality assessment of studies between the authors were discussed with the senior author (GH).

### Data Synthesis

Although, it was intended to conduct a meta-analysis, this was not possible due to the heterogeneity between studies surrounding MM definitions (and therefore outcomes), study designs, reporting of results, and statistical methods. Thus, a narrative synthesis was conducted, which evaluated the strength of the evidence and discussed how the factors can be organised thematically into personal traits, micro-, meso-, and macro-levels. The strength of the evidence for each factor was determined based on the number of studies for each identified factor, the consistency of the findings, and the study quality (from JBI scores). The narrative synthesis was guided by recommendations from Popay et al.^37^ The ROBIS tool^38^ was used to assess risk of bias (Table S10) and any concerns were addressed in subsequent revisions of the synthesis.

## Results

**Figure 1:**
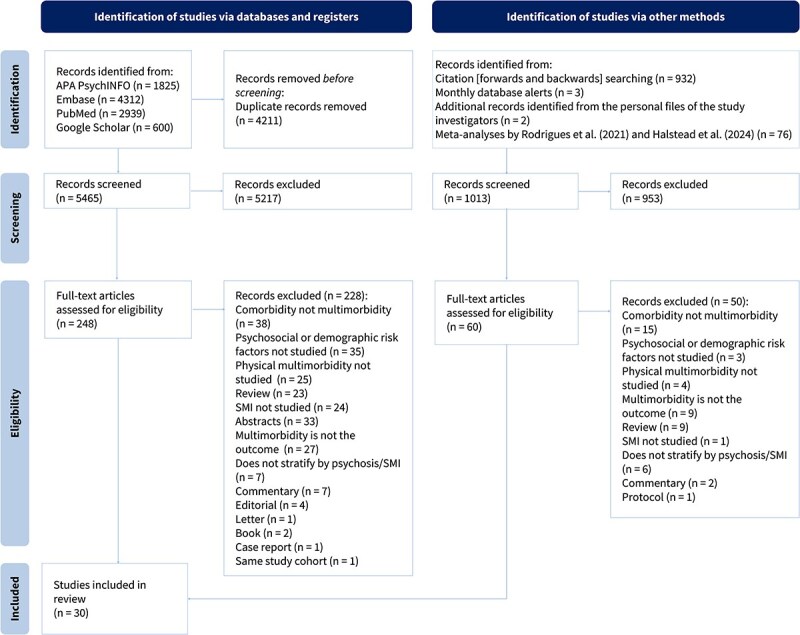
Preferred Reporting Items for Systematic Reviews and Meta-Analysis Flow Diagram Outlining the Study Selection Process.

The initial search identified 5465 studies (Figure 1). Full texts of 308 studies were reviewed for eligibility and 30 studies met the inclusion criteria for this review. Reasons for exclusion are listed in Figure 1. All 30 articles investigated psychosocial or demographic associations with MM outcomes among individuals with SMI.

A summary of the study characteristics is presented in Table 1. Studies took place in the United Kingdom (*n* = 9),^2^^,^^9^^,^^25^^,^^39–44^ Europe (*n* = 5),^45–49^ North America (*n* = 5),^50–54^ Asia (*n* = 4),^55–58^ Africa (*n* = 2),^59^^,^^60^ Australasia (*n* = 2),^61^^,^^62^ and the Middle East (*n* = 1).^63^ Two studies were conducted across multiple countries.^64^^,^^65^ Most studies were of cross-sectional design (*n* = 18),^2^^,^^9^^,^^43^^,^^44^^,^^46–50^^,^^53^^,^^55^^,^^58–60^^,^^62–65^ followed by cohort (*n* = 6),^25^^,^^41^^,^^42^^,^^52^^,^^54^^,^^57^ case-control (*n* = 2),^39^^,^^56^ observational (*n* = 2),^51^ prevalence (*n* = 1),^40^ and retrospective designs (*n* = 1).^61^ Sample sizes across the studies ranged from 78^59^ to 31 807 participants with SMI.^42^

**Table 1:** Study Characteristics of the Included Papers

**Study country**	**Study design**	**SMI disorder diagnoses**	**Definition of MM**	**Sample size (*n*)**	**Mean age of cases in years (SD)** **Age range (years)**	**Gender/sex of cases (%)**	**Ethnicity of cases (%)**	**Measure of SMI**	**Care setting**	**Psychiatric comorbidity (Y/N)**	**Medication (Y/N)**
Bouza et al. (2010) Spain	Observational retrospective study	Schizophrenia	Number of physical ICD-9 codes	16 776	43.5 (16.0) 15-85+	Male 65.0 Female 35.0	NR	ICD-9 Codes 295.xx	Acute and scheduled hospital admissions	NR	NR
Charlson et al. (2021) Australia	Retrospective study	Any of the below psychotic disorders: Organic-related Substance use-related Schizophrenia-related Acute and transient psychoses Mood disorder-related	Number of comorbid physical disorders: 2, 3, or more	426	NR	Male 65.7 Female 34.3	Aboriginal 60.8 Torres Strait Islander 30.8	ICD-10 Codes F06, F10-19, F20-22, F23, F25, F30-33	Outpatient	Y	NR
de Freitas et al. (2022) United Kingdom	Retrospective cohort study	SSD	3 categories of multimorbidity severity, in addition to psychosis: 1 or 2 physical health conditions 3 or more physical health conditions	20 800	Median 36.9; IQR 27.0-47.1 13-65	Male 60.5 Female 39.5	White British 35.0 Black British/Other Black background 15.1 Black African 13.7 Black Caribbean 8.8 Other White background 8.6 Asian British/Other Asian background 4.1 Mixed ethnic background 3.3 Irish 1.6 Indian 1.6 Pakistani 1.0 Chinese 0.6 Bangladeshi 0.6 Other ethnic background 6.1	ICD-10 Codes F20-F29	NR	NR	NR
Dixon et al. (1999) United States	Cross-sectional study	Schizophrenia	Number of current medical conditions alongside schizophrenia	719	43.2 (12.0) 18+	Male 63.0 Female 37.0	White 54.0 African American/Asian/Other 46.0	NR	Inpatient and outpatient	Y	Y
Domino et al. (2014) United States	Retrospective cohort study	Schizophrenia	Number of non-psychiatric medical conditions (2 conditions; 3 or more)	15 636	Total sample mean 43.0 (13.0)	Male 47.1 Female 52.9	African American 53.3 Latino 1.9 Unspecified 44.8	Medicaid claim	Inpatient and outpatient	Y	Y
Fenn et al. (2005) United States	Cross-sectional study	Bipolar I Bipolar II	Number of medical comorbidities	330	45.9 (10.0) ≤30-71+	Male 91.0 Female 9.0	NR	DSM-IV	Inpatient and outpatient	Y	NR
Filipcic et al. (2019) Croatia	Nested cross-sectional study	SSD	2 or more chronic physical illnesses	329 SSD 837 controls	Men 41 (12.5) Women 45 (13.0) 18+	Men 55.9 Women 44.1	NR	ICD-10 Codes F20-F29	Inpatient and outpatient	NR	Y
Gabilondo et al. (2017) Basque Country, Spain	Retrospective cross-sectional study	Schizophrenia	Multiple comorbidities: co-occurrence of 2 or more health problems in a person with schizophrenia	7331 schizophrenia 2 248 075 controls	48.6	Male 60.4 Female 39.6	NR	ICD-10 F20	NR	NR	NR
García-Goñi et al. (2021) Basque Country, Spain	Cross-sectional study	Schizophrenia Affective psychosis Bipolar disorder	The presence of at least one additional chronic disease to the existing mental health condition Number of chronic conditions alongside mental health condition (0 to 10+)	15 141 schizophrenia, affective psychosis or bipolar disorder 2 262 698 total sample	Mean NR Range 0-75	Male 53.3 Female 46.7	NR	ICD-9-CM	Inpatient and outpatient	Y	NR
Godin et al. (2023) France	Cross-sectional study	Bipolar disorder (All bipolar subtypes [I, II, not otherwise specified])	Medical morbidity was defined by the sum of medical disorders	2891	40.5 (12.9) 16+	Male 37.8 Female 62.2	NR	DSM-IV	Outpatient	Y	Y
Hosang et al. (2018) United Kingdom	Case-control study	Bipolar disorder	Number of medical illnesses (1, 2 or more)	72 Bipolar disorder 354 controls	48.4 (9.4) 18+	Women 78.0	All participants were White	DSM-IV	Outpatient	NR	NR
Hsu et al. (2021) Taiwan	Retrospective cohort study	Schizophrenia	CCI and the number of comorbidities	3827	72.9 (6.4) 65+	Male 52.4 Female 47.6	NR	ICD-9	Inpatient and outpatient	NR	NR
Jahrami et al. (2017) Bahrain	Case-control study	Schizophrenia of any type	Number of physical health comorbidities (1, 2 or ≥3 comorbidities)	120 schizophrenia 120 matched controls	41.69 (13) 20-60	Male 55.0 Female 45.0	NR	ICD-10	Outpatient	NR	Y
Lasebikan and Azegbeobor (2017) Nigeria	Cross-sectional study	Schizophrenia Bipolar I disorder	Multiple medical comorbidity (≥3 general medical conditions)	1310 schizophrenia 1307 bipolar I disorder 1310 controls	Schizophrenia: Median 30.0 Bipolar I disorder: Median 34.0 18+	Schizophrenia: Male 51.9 Female 48.1 Bipolar I disorder: Male 55.2 Female 44.8	NR	DSM-IV	Inpatient	NR	NR
Launders et al. (2022) United Kingdom	Cohort-nested accumulated prevalence study	Schizophrenia Bipolar disorder Other non-affective psychotic illnesses	2 or more physical health conditions	68 783 SMI 274 684 no SMI	50.67 (19.06) 18-80+	Female 49.0	Asian 4.7 Black 5.3 Mixed 1.2 Other 2.2 White 86.6	Read codes from primary care databases	Primary care	NR	NR
Mirabzadeh et al. (2020) Iran	Cross-sectional study	Schizophrenia	Number of non-psychiatric disorders	395	Mean NR	Male 68.9 Female 31.1	NR	DSM-5	Inpatient	NR	Y
Mirza et al. (2021) United Kingdom	Cross-sectional study	SSD Bipolar disorder	Complex multimorbidity: having 2 or more organ systems affected besides the SMI diagnoses	13 933	Mean NR 15-75+	Male 52.2 Female 47.8	British White 34.1 Black African 13.3 Black Caribbean 8.8 South Asian 3.4 Irish White 2.1 Chinese 0.7 Unknown 37.5	ICD-10 F20-29; F30-31	Inpatient, outpatient, and community services	Y	NR
Monk et al. (2024) New Zealand	Cross-sectional study	Schizophrenia Bipolar disorder type 1 Depression with psychosis Substance-induced psychosis Schizoaffective disorder Other Organic psychosis Not otherwise specified psychosis	M3 multimorbidity index	21 214	Māori 38.1 (11.9) Non-Māori 41.9 (12.5) 16-64	Male 59.0 Female 41.0	Māori 34.3 Non-Māori 65.7	DSM-IV ICD-10	Inpatient and outpatient	Y	Y
Owen et al. (2023) United Kingdom	Retrospective cohort study	Psychotic symptoms SSD Bipolar disorder	Coexistence of 2 or more long-term health conditions in an individual	1 675 585 total sample As the index condition, 24 990 individuals developed psychosis	Total sample: Median 51.0 (IQR 37.0-65.0)	Total sample: Men 48.4 Women 51.6	NR	ICD-10 F20-29; F30-31 Elixhauser Read Codes	Inpatient and outpatient	NR	NR
Post et al. (2013) United States, The Netherlands and Germany	Cross-sectional study	Bipolar I Bipolar II Schizoaffective disorder, bipolar type	Number of medical comorbidities (none, few [1-3], many [4 or more])	904 completed all questions relating to childhood adversity burden (*n* = 890 in most analyses)	41.0 Age range NR	NR	NR	Previous clinical diagnosis Validated by SCID interview	Outpatient	NR	NR
Public Health England (2018) United Kingdom	Cross-sectional study	Schizophrenia Affective disorder (bipolar or unspecified affective disorder) Other types of psychoses	More than one physical health condition at the same time as their mental illness (Data extracted for 3 or more conditions)	1 051 127 in database 9357 with SMI diagnosis	Mean NR 15-74	Male 52.3 Female 47.7	NR	Quality and Outcomes Framework (QOF) business rules using Read Codes	Primary care	NR	Y
Reilly et al. (2015) United Kingdom	Retrospective cohort study	Schizophrenia Affective disorder (bipolar or unspecified affective disorder) Other types of psychoses	Number of additional conditions to SMI diagnosis	For 2011/12: 31 807 SMI 159 035 Controls	51.6 (16.7) 18+	Male 48.9	NR	Read Codes from the Clinical Practice Research Datalink (CPRD)	Primary care	Y	Y
Rodrigues et al. (2022) Canada	Matched retrospective cohort study	Schizophrenia Schizoaffective disorder Delusional disorder Psychosis not otherwise specified	Co-occurrence of 2 + chronic health conditions from a list of 17 conditions, which included 16 physical conditions and 1 mental health condition	439 people with psychotic disorders 1759 matched controls	Mean NR 16-33	Male 75.2 Female 24.8	NR	ICD-9 and ICD-10	Inpatient and outpatient	Y	NR
Rojanaworarit et al. (2025) Thailand	Cross-sectional study	Schizophrenia	Co-occurrence of at least one systemic disease in addition to schizophrenia	231	47.4 (11.5) 20-98	Male 100	NR	DSM-IV and DSM-5	Patients experiencing homelessness in an aid center	Y	NR
Smith et al. (2013a) Scotland, United Kingdom	Cross-sectional study	Schizophrenia or related non-organic psychosis	Comorbidities: Presence of 2 or 3 or more physical health comorbidities	9677 people with psychotic disorders 1 414 701 controls	51.6 (16.5) 18+	Male 51.3 Female 48.7	NR	Primary care read codes	Primary care	NR	NR
Smith et al. (2013b) Scotland, United Kingdom	Cross-sectional study	Bipolar disorder	Comorbidities: Presence of 2 or 3 or more physical health comorbidities	2582 people with bipolar disorder 1 421 796 controls	54.5 (15.3) 18+	Male 39.5 Female 60.5	NR	Primary care read codes	Primary care	NR	Y
Stapp et al. (2020) United States	Observational study	Mood disorder: Depression Dysthymia Mania/Hypomania (Bipolar disorder)	Medical morbidity: Number of past-year medical conditions	7621 mood disorder (including mania and hypomania) 27 032 no mood disorder	Mean NR 20+	Total sample: Men 47.9 Women 52.1	Total sample: White Non-Hispanic 70.9 Black Non-Hispanic 11.0 American Indian/Alaskan Native 2.2 Asian/Hawaiian/Pacific Islander 4.3 Hispanic 11.6	DSM-IV Diagnostic interview used was AUDADIS-IV	Outpatients	NR	NR
Stubbs et al. (2016) 48 low- and middle-income countries (LMICs)	Cross-sectional study	Subclinical psychosis (at least one psychotic symptom but no psychosis diagnosis) Psychosis (schizophrenia or psychosis diagnosis)	2 or more physical health conditions	25 493 subclinical psychosis 2224 psychosis diagnosis 179 429 controls	Total sample: 38.4 (16.0) 18+	Total sample: Male 49.4 Female 50.6	NR	Self-report WHO Composite International Diagnostic Interview (CIDI)	NR	NR	NR
Teh et al. (2021) Singapore	Cross-sectional study	Schizophrenia Non-affective psychotic disorders: Schizoaffective disorder Schizophreniform disorder Brief psychotic disorder Delusional disorder Psychotic disorder NOS Affective psychotic disorders: Bipolar I disorder Major Depressive Disorder and with psychotic features	Number of co-occurring chronic medical/physical condition in addition to the index condition of psychotic disorders	364	35.2 21-65	Male 46.0 Female 53.7	Chinese 69.0 Malay 15.6 Indian 11.0 Others 4.1	DSM-IV	Outpatient	NR	NR
Thabet et al. (2019) Tunisia	Retrospective descriptive study	Schizophrenia Schizoaffective disorder	Number of somatic comorbidities	78	41.8 (11.6) 21-65	Men 79.6 Women 23.1	NR	DSM-IV	Outpatient	Y	Y

Abbreviations: CCI, Charlson Comorbidity Index; MM, multimorbidity; NOS, not otherwise specified; NR, not reported; SMI, severe mental illness; SSD, schizophrenia spectrum disorder; DSM-IV, Diagnostic and Statistical Manual of Mental Disorders, Fourth Edition; DSM-5, Diagnostic and Statistical Manual of Mental Disorders, Fifth Edition.

### Study Quality

Study quality was rated high across most studies (*n* = 20) and moderate in 10 studies (Table S6). Of the moderate quality studies, six employed a cohort design and four used cross-sectional designs. Reasons for study quality meeting “moderate” criteria included insufficient information on study settings and analytic methods, small sample sizes and lacking consideration of confounding factors.

### Case Definitions and Measures of SMI

Study samples were restricted to schizophrenia or other psychotic disorders in 14 studies.^25^^,^^43^^,^^45^^,^^46^^,^^48^^,^^50^^,^^52^^,^^54^^,^^56–59^^,^^62^^,^^63^ Ten studies investigated individuals with SMI (schizophrenia, related psychotic disorders and bipolar disorder),^2^^,^^9^^,^^40–42^^,^^47^^,^^55^^,^^60^^,^^61^^,^^65^ and four studies examined MM among individuals with bipolar disorder.^39^^,^^44^^,^^49^^,^^53^ One study included both individuals with psychotic disorders or subclinical psychosis,^64^ and another investigated those with mania/hypomania to explore differences in episode polarity in bipolar disorder.^51^ Most studies used the ICD (9th or 10th edition) as the diagnostic measure for SMI (*n* = 12).^9^^,^^25^^,^^41^^,^^45–48^^,^^52^^,^^56^^,^^57^^,^^61^^,^^62^ Other measures of SMI included the Diagnostic and Statistical Manual of Mental Disorders, Fourth or Fifth Edition ([DSM-IV, DSM-5], *n* = 9),^39^^,^^49^^,^^51^^,^^53^^,^^55^^,^^58–60^^,^^62^ read codes from primary care databases (*n* = 5),^2^^,^^40^^,^^42–44^ diagnostic interviews (*n* = 2),^64^^,^^65^ and claims diagnoses (*n* = 1)^54^ (Table 1).

### Definition of Multimorbidity

Studies used varied definitions of MM. Some studies looked at the total or average number of comorbid conditions,^42^^,^^45^^,^^47^^,^^49–51^^,^^53–55^^,^^59^^,^^63^ while others adopted threshold criteria for MM, whereby a person had MM if they were diagnosed with one or more, two, or three or more chronic health conditions alongside their SMI diagnosis.^2^^,^^9^^,^^25^^,^^39–41^^,^^43^^,^^44^^,^^46^^,^^48^^,^^52^^,^^56^^,^^58^^,^^60^^,^^61^^,^^65^ Two studies used MM indices, such as the Charlson Comorbidity Index (CCI)^57^ and the M3 Multimorbidity Index,^62^ where scores are weighted by the predictiveness of mortality for each comorbid condition.

Most studies investigated the existence of comorbid diagnoses from a predefined list of chronic conditions, mainly cardiovascular disease, diabetes, respiratory diseases, cancer, and musculoskeletal disorders. A small number of studies considered physical illnesses and the co-occurrence of common mental illnesses^52^ outside of the SMI (e.g. mood or anxiety disorder), or learning disabilities.^42^ One study included abuse or dependency on drugs, alcohol or tobacco in their definition of MM^45^. The co-occurrence of specific illnesses (i.e. diabetes and congestive heart failure) within this population was investigated in one study^41^ (Table S7).

### Factors Associated With Physical Multimorbidity in SMI

A total of 13 psychosocial and demographic factors associated with MM were identified in the included articles, but with mixed evidence. These were: gender/sex, age, ethnicity, educational attainment, employment status, marital status, place (e.g. urbanicity and country/state of origin), socioeconomic status, deprivation, childhood maltreatment, healthcare setting, and global functioning (Table 2). Factors with the strongest evidence for their associations with MM in SMI were female gender/sex, older age, childhood maltreatment, and ethnicity.

**Table 2:** Summary of Psychosocial and Demographic Factors Identified With At-Risk Groups

**Psychosocial and demographic factors identified**	**At-risk groups**	**Number of studies with supporting evidence (total studies)**
*Factors with the strongest evidence*		
Gender/sex	Women/female sex	10 (20)
Age	Older age	12 (17)
Childhood maltreatment	Experiences of childhood abuse and neglect	4 (4)
Ethnicity	Black African, Black Caribbean, and Black British groups (relative to White British groups) Malay and Indian groups (relative to Chinese ethnic groups) Aboriginal (relative to Torres Strait Islanders) Māori (relative to non-Māori)	4 (7)
*Factors with mixed evidence*		
Deprivation	Residents in areas with the highest levels of multiple, material or area-level deprivation	4 (6)
Education	Those without university-level education	2 (5)
Employment status	Unemployment	1 (2)
Marital status	Being married	2 (4)
Socioeconomic status	Lower socioeconomic groups	1 (2)
Healthcare setting	Public healthcare users (relative to private healthcare)	1 (1)
Country or state of residence	United States (relative to the Netherlands and Germany)	1 (3)
Urbanicity	Residents in urban areas	1 (2)
Functioning	Those with poorer global functioning scores	1 (1)

The total number of studies listed in each cell does not equal the total number included in the review, as not all studies investigated every factor.

Clinical, health, and lifestyle factors were commonly reported in the included studies. This information was collated and synthesised to provide a comprehensive picture of the known risk factors for MM in SMI (Tables S4 and S5). Clinical factors included age of onset and duration of SMI illness, number of episodes, hospitalisations, attempted suicide, polypharmacy, antipsychotic treatment, other medication, intellectual or physical disabilities, poor functioning, rapid cycling, psychiatric symptoms, SMI diagnosis, presence of anxiety disorder, and sleep disturbances. Health and lifestyle factors included tobacco smoking, substance abuse, alcohol intake, increased/decreased weight, physical inactivity, and excessive caloric intake.

#### Demographic and Psychosocial Factors

Table 3 presents an overview of the demographic and psychosocial factors associated with MM. Study effect sizes are presented in Tables S2 and S3. Findings across the studies were mixed, though this narrative synthesis attempts to order the findings based on the strength of evidence.

**Table 3:** Demographic and Psychosocial Factors Associated With Multimorbidity in the Included Studies

**Study**	**Female gender/sex**	**Older age**	**Ethnicity**	**Low educational level**	**Unemployment**	**Being married**	**Urbanicity**	**Low socioeconomic standards**	**High deprivation**	**Childhood adversity**	**Country or state of origin**	**Healthcare setting (public vs private)**	**Poorer functioning**
Bouza et al. (2010)	↑	-	-	-	-	-	-	-	-	-	-	-	-
Charlson et al. (2020)	X^a^	-	↑ Aboriginal (relative to Torres Strait Islanders)^a^	-	-	-	-	-	-	-	-	-	-
de Freitas et al. (2022)	↓^b^ 3 or more physical health conditions	↑^b^	↑^b^ Black African, Black Caribbean, and Black British groups (rel. to White British) ↓^b^ Other White Background, Chinese, Other ethnic background (rel. to White British)	-	-	-	-	-	↑^b^ Multiple deprivation	-	-	-	-
Dixon et al. (1999)	↑	↑	X	X	-	↑	X	-	-	-	X Southern vs Midwestern US states	↑	-
Domino et al. (2014)	↑^a^	-	↓ Latino and African American (relative to ethnicity unspecified)^a^	-	-	-	-	-	-	-	-	-	-
Fenn et al. (2005)	-	↑	-	-	-	-	-	-	-	-	-	-	-
Filipcic et al. (2019)	↑^b^	X^b^	-	↑ Primary vs University education^b^ X Primary vs Secondary education^b^	X^b^	X^b^	-	-	-	-	-	-	-
Gabilondo et al. (2017)	↑^a^	-	-	-	-	-	-	-	-	-	-	-	-
García-Goñi et al. (2021)	-	-	-	-	-	-	-	↑ Socioeconomic inequality^b^	-	-	-	-	-
Godin et al. (2023)	↑	↑	-	↑ No Bachelor degree vs Bachelor degree	-	-	-	-	-	↑Overall childhood maltreatment^b^ ↑ Childhood abuse (abuse + neglect)^b^ ↑ Sexual abuse^b^	-	-	-
Hosang et al. (2018)	-	-	-	-	-	-	-	-	-	↑ Overall childhood maltreatment^b^ ↑ Childhood abuse^b^ ↑ Childhood neglect^b^	-	-	-
Hsu et al. (2021)	↓^a^	↑^a^	-	-	-	-	-	-	-	-	-	-	-
Jahrami et al. (2017)	X	↑	-	-	-	-	-	-	-	-	-	-	-
Lasebikan and Azegbeobor (2017)	-	-	-	-	-	-	-	-	-	-	-	-	↑ Poorer global assessment of functioning
Launders et al. (2022)	↑^a^	↑^a^	-	-	-	-	-	-	-	-	-	-	-
Mirabzadeh et al. (2020)	-	↑^a^	-	-	-	-	-	-	-	-	-	-	-
Mirza et al. (2021)	X	-	X	-	-	-	-	-	X Multiple deprivation	-	-	-	-
Monk et al. (2024)	-	-	↑ Māori (relative to non-Māori)^a^	-	-	-	-	-	-	-	-	-	-
Owen et al. (2023)	X^a^	-	-	-	-	-	-	-	↑ Multiple deprivation^a^	-	-	-	-
Post et al. (2013)	↑ 1-3 medical comorbidities vs none^b^ ↑ 4+ medical comorbidities vs having 1-3 comorbidities^b^	↑ 1-3 medical comorbidities vs none^b^ X 4+ medical comorbidities vs having 1-3 comorbidities^b^	-	-	-	-	-	-	-	↑ 1-3 medical comorbidities vs none^b^ ↑ 4+ medical comorbidities vs having 1-3 comorbidities^b^	X 1-3 medical comorbidities vs none^b^ ↑ United States (for 4+ medical comorbidities vs having 1-3) relative to European countries^b^	-	-
Public Health England (2018)	X^b^	↑^b^	-	-	-	-	-	-	X Material deprivation^b^	-	-	-	-
Reilly et al. (2015)	-	-	-	-	-	-	-	-	↑ Bipolar disorder and unspecified/ other affective psychosis^a^ X All SMI diagnoses, schizophrenia, and other types of psychosis^a^	-	-	-	-
Rodrigues et al. (2022)	X	X	-	-	-	-	-	-	↑ Material deprivation	-	-	-	-
Rojanaworarit et al. (2025)	-	X^b^	-	-	-	-	-	-	-	-	-	-	-
Smith et al. (2013a)	↑	-	-	-	-	-	-	-	-	-	-	-	-
Smith et al. (2013b)	↑ 3 or more conditions X 2 or more conditions	-	-	-	-	-	-	-	-	-	-	-	-
Stapp et al. (2020)	-	-	-	-	-	-	-	-	-	↑ Sexual abuse^b^ ↑ Emotional abuse^b^ X Physical abuse^b^ X Physical neglect^b^ X Emotional neglect^b^	-	-	-
Teh et al. (2021)	X	↑	↑ Malay and Indian groups (relative to Chinese ethnic groups)	X	-	X	-	-	-	-	-	-	-
Thabet et al. (2019)	↓	↑	-	X	↑	↑	↑	X	-	-	-	-	-

↑ Positive association between factor and prevalence/odds/incidence of MM.

↓ Inverse association between factor and prevalence/odds/incidence of MM.

X No association between factor and physical MM.

- Association not studied.

a Statistical significance was calculated using information available in the paper.

b Association adjusted for other factors/covariates.

Material deprivation is based on factors such as average neighbourhood-level income, educational attainment, family structure, quality of housing, car ownership, overcrowding, unemployment, and low social class. Multiple deprivation includes these factors plus broader concepts such as crime, health, access to housing and services, and living environment. Abbreviation: SMI, severe mental illness.

##### Factors With the Strongest Evidence: Gender/Sex

Twenty studies investigated gender or sex differences in MM among people with SMI.^2^^,^^9^^,^^25^^,^^40^^,^^41^^,^^43–46^^,^^48–50^^,^^52^^,^^54–57^^,^^59^^,^^61^^,^^65^ Seven studies investigated gender,^25^^,^^43–45^^,^^48^^,^^50^^,^^65^ 10 investigated sex,^2^^,^^9^^,^^40^^,^^41^^,^^49^^,^^52^^,^^54^^,^^56^^,^^57^^,^^61^ and three studies used the terms interchangeably.^46^^,^^55^^,^^59^ However, differences in the conceptualisation of gender and sex did not meaningfully affect the synthesis. The evidence surrounding gender/sex in MM is mixed. Ten studies found that female gender/sex was associated with increased odds of MM, higher MM prevalence rates, or a greater number of comorbidities across SMI diagnoses.^40^^,^^43–46^^,^^48–50^^,^^54^^,^^65^ Women were found to be up to 2.37 times more likely to have MM than men, even after controlling for other sociodemographic factors in one study.^48^

In contrast, three studies found the opposite effect of gender/sex, where men were more likely to have three or more physical health conditions relative to women,^25^ a higher CCI score^57^ and more comorbidities,^59^ though effect sizes in this direction were small. Seven studies did not observe any significant gender or sex differences in MM outcomes in SMI populations.^2^^,^^9^^,^^41^^,^^52^^,^^55^^,^^56^^,^^61^ Variations in MM definitions and statistical approaches may partly explain the inconsistent findings. Four of the 20 studies controlled for confounding variables (e.g. age) in their analyses, suggesting a potential risk of bias in the remaining studies.^2^^,^^25^^,^^48^^,^^65^ Studies that found increased MM in women mostly conceptualized MM using threshold criteria (i.e. diagnosis of two or more conditions), while those that found higher MM rates among men used heterogeneous definitions.

##### Age

Seventeen studies investigated the association between age and MM outcomes in SMI.^2^^,^^25^^,^^40^^,^^45^^,^^48–50^^,^^52^^,^^53^^,^  ^55–59^^,^^63–65^ Twelve studies reported older age to be significantly associated with increased MM risk or more comorbidities.^2^^,^^25^^,^^40^^,^^49^^,^^50^^,^^53^^,^^55–57^^,^^59^^,^^63^^,^^65^ For example, people aged 50-65 years old were 1.53 times more likely to have three or more physical health conditions relative to those aged 13-17 years.^25^ However, five of these studies presented a moderate risk of bias and did not control for confounding factors.^50^^,^^53^^,^^57^^,^^59^^,^^63^ Half of the studies treated age as a continuous variable,^48^^,^^53^^,^^55^^,^^56^^,^^59^^,^^65^ while other studies generally defined older age as over 40 or 50 years.^2^^,^^25^^,^^49^^,^^50^ Two additional studies reporting mixed effects did not conduct inferential analyses, so significance could not be determined based on the information available.^45^^,^^64^

One study investigated the interaction between gender and age on MM prevalence. Age was not associated with MM, but MM rates were significantly higher among younger women but not men with schizophrenia spectrum disorder relative to controls.^48^

##### Childhood Maltreatment

Four studies investigated the impact of childhood maltreatment on MM outcomes in bipolar disorder.^39^^,^^49^^,^^51^^,^^65^ No studies explored the impact of childhood maltreatment on MM outcomes in other SMI diagnoses. Childhood maltreatment, defined as experiences of abuse and neglect during childhood and adolescence, was consistently found to be associated with increased MM risk.^39^^,^^49^^,^^51^^,^^65^ Childhood abuse and neglect increased the odds of MM by up to eight times.^39^ These effects persisted after adjusting for potential confounders (age, gender, illness duration). This association showed a dose-response relationship: two or more forms of childhood maltreatment showed the highest odds of MM relative to those without any history of childhood maltreatment or those who experienced one form.^39^ Similarly, higher childhood adversity scores were shown to be the strongest predictor of having many medical comorbidities (i.e. 4 or more) relative to having fewer comorbidities (1-3 conditions).^65^

Two studies examined the impact of specific forms of childhood maltreatment (e.g. emotional and sexual abuse) on MM risk.^49^^,^^51^ Emotional abuse, emotional neglect, and sexual abuse were significantly associated with medical morbidity in one study.^49^ In another, sexual and emotional abuse were associated with more medical conditions among people with manic and hypomanic symptoms.^51^

##### Ethnicity

Of seven studies examining ethnicity and MM outcomes among individuals with SMI, ^9^^,^^25^^,^^50^^,^^54^^,^^55^^,^^61^^,^^62^ four reported that minoritised ethnic groups were at the greatest risk of physical MM.^25^^,^^55^^,^^61^^,^^62^ All four studies were of high quality. In the United Kingdom, MM was more common in Black African, Black Caribbean and Black British groups (relative to White British groups)^25^; increased odds of severe MM (three or more physical health conditions) were observed for individuals of any Black background relative to White British groups (Black British AOR = 2.06, 95% CI 1.83-2.31; Black African AOR = 1.50, 95% CI 1.33-1.70; Black Caribbean AOR = 2.09, 95% CI 1.81-2.42).^25^ In Singapore, Malay and Indian groups had significantly greater odds of MM relative to Chinese ethnic groups,^55^ and significantly higher rates of MM were also observed in Aboriginal patients relative to Torres Strait Islander patients in Cape York and the Torres Strait (Australia).^61^ In New Zealand, Māori individuals with psychosis had a significantly higher mean M3 (MM) score than non-Māori individuals, though this effect size was small.^62^

Conversely, a moderate quality study in North Carolina found that Latino people with schizophrenia had significantly lower MM rates relative to those whose ethnicity was unspecified, and there were no significant differences in MM rates between those from African American and unspecified backgrounds.^54^ However, the ‘unspecified’ ethnic group may have also captured individuals of minoritised ethnicities, which questions the finding’s reliability.

In a UK study, reduced odds for MM were observed among Other and Chinese backgrounds (relative to White British groups).^25^ This study went one step further and explored intersectional effects, specifically whether the impact of ethnicity differed by gender or deprivation level, but no significant effects were observed.^25^ Two studies did not observe any ethnic differences in MM outcomes.^9^^,^^50^

#### Other Psychosocial Factors: Deprivation

Six studies assessed the impact of deprivation.^2^^,^^9^^,^^25^^,^^41^^,^^42^^,^^52^ Deprivation indices were operationalised differently across studies. Some focused on material deprivation which accounts for multiple facets such as housing quality, unemployment, educational attainment, and neighborhood-level income (e.g. Ontario Marginalization Index^52^). Other studies investigated multiple deprivation (e.g. Index of Multiple Deprivation), which includes broader concepts such as crime, health, and access to housing and services.^9^^,^^21^^,^^41^^,^^66^ Deprivation was significantly associated with MM in four studies; residing in areas with the highest levels of multiple, area-level, or material deprivation was associated with higher MM rates among SMI groups in the United Kingdom^25^^,^^41^^,^^42^ and Canada.^52^ However, three of these cohort studies presented a moderate risk of bias since methods were not clearly reported to address incomplete follow-up. The remaining two UK studies found no associations between deprivation and MM.^2^^,^^9^ Varied operationalisation of deprivation across studies may in part explain these mixed findings.

#### Education

Five studies investigated the association between education (defined either as the highest level of educational attainment or number of years in education) and MM and report disparate results. Two studies conducted in Croatia and France reported university-level education and having a Bachelor’s degree as protective factors against MM.^48^^,^^49^ Three studies (conducted in Singapore, Tunisia, and the United States) did not observe any significant associations between educational attainment and MM outcomes among SMI groups,^50^^,^^55^^,^^59^ though two of these presented moderate risk of bias. Inconsistencies in the findings may be attributed to differences in educational standards and practices across countries. The impact of educational attainment on MM outcomes therefore remains unclear.

#### Employment Status

Two studies investigated employment status and MM^48^^,^^59^ with inconsistent results. One study reported more physical health comorbidities among unemployed individuals with schizophrenia relative to those who were employed,^59^ however the sample consisted of only 78 participants. These findings were not replicated in another study.^48^ In the first sample, a larger proportion of participants were unemployed relative to employed (75.6% vs 24.4%),^59^ whereas in the second sample, more participants were employed (40.2% vs 28.2% unemployed and 31.6% retired).^48^ This, along with varying sample sizes, may partly explain the mixed findings observed.

#### Marital Status

Four studies investigated the association between marital status and MM^48^^,^^50^^,^^55^^,^^59^ and reported disparate patterns. Most participants were single in three studies (up to 75% of each sample, relative to being married or widowed).^48^^,^^55^^,^^59^ This information was not available in another study.^50^ Two studies found that being married was significantly associated with a higher number of comorbidities among individuals with SMI.^50^^,^^59^ However, these studies presented moderate risk of bias; one used a small sample^59^ and the other used self-reported measures of physical health diagnoses.^50^ The remaining two studies reported similar findings, but these were not statistically significant.^48^^,^^55^ Studies did not explore the quality of marital relationships (e.g. tension) that might contribute to MM.

#### Socioeconomic Status

Two studies investigated the impact of socioeconomic status on MM^47^^,^^59^ with inconsistent results. One study observed a positive association between socioeconomic inequality (i.e. inequality in mental health being attributed to socioeconomic status using a concentration index) and the number of comorbidities.^47^ Another study did not observe a significant relationship between socioeconomic standards and MM,^59^ though this study was of moderate quality where details of how socioeconomic standards were measured were not reported.

#### Healthcare Setting

Only one study investigated the association between healthcare setting and MM outcomes. In the United States, those recruited from public healthcare settings reported more comorbidities than private healthcare users. ^50^ However, as this study presented a moderate risk of bias (e.g. used a self-report comorbidities measure) the findings should be treated with caution.

#### Place

The factor ‘place’ encompasses areas of residence by country, state, or level of urbanicity. Three studies examined differences in MM outcomes based on the country or US state of residence.^50^^,^^64^^,^^65^ Only one study observed an association, where individuals with bipolar disorder in the United States were more likely to report having four or more comorbidities (relative to 1-3) than those in the Netherlands or Germany. ^65^ This effect size was small, and the association was not significant when comparing those with 1-3 comorbidities to those with none. No significant differences were observed in the studies that investigated differences in MM outcomes among people with psychosis across Midwestern and Southern US States^50^ or between low- and middle-income countries,^64^ though it was not possible to make statistical inferences in this study based on the information available.

Two studies investigated the impact of urbanicity. One reported a significant positive relationship between residing in urban areas and the number of comorbidities,^59^ while another reported no significant differences in the number of comorbidities between rural and urban sites.^50^ Both studies were of moderate quality with significant limitations such as a small sample size of 78 participants.^59^

#### Functioning

Only one study investigated the relationship between social, occupational, and psychological functioning and MM risk in schizophrenia and bipolar disorder. Individuals with poorer functioning scores (as measured by the Global Assessment of Functioning Scale) had greater odds of MM than those with higher functioning scores.^60^

### Clinical, Health and Lifestyle Factors

Clinical, health, and lifestyle factors investigated in relation to MM are presented in Tables S4 and S5, with effect sizes listed in Tables S2 and S3. These are factors that were highlighted in the included papers, but were not systematically derived findings.

#### SMI Diagnosis

Seven studies investigated differences in MM outcomes across SMI diagnoses,^9^^,^^40^^,^^42^^,^^48^^,^^49^^,^^55^^,^^64^ and reported mixed results. Three studies found that individuals diagnosed with schizophrenia were more likely to report MM than bipolar disorder,^9^ other affective and non-affective psychotic disorders,^55^ or subclinical psychosis.^64^ Conversely, one study found that individuals with bipolar disorder or other SMI diagnoses reported significantly higher rates of MM than individuals with schizophrenia.^40^ Another study observed significant differences in the mean number of comorbid conditions between bipolar disorder subtypes, where those with bipolar disorder I had fewer comorbid conditions than those with bipolar disorder II or not otherwise specified, though this difference was marginal.^49^ Similarly, another study found that individuals with unspecified or other affective psychosis reported significantly more physical comorbidities than other SMI diagnoses.^42^ One study did not observe any differences in MM outcomes across SMI diagnoses.^48^

#### Age of SMI Onset and Duration

Six studies investigated age of onset and illness duration, where four reported significant associations between earlier age of SMI onset/diagnosis^9^^,^^55^ and longer illness duration^49^^,^^60^ with MM. Two studies failed to replicate these results.^48^^,^^52^

#### SMI Symptoms

Four studies investigated SMI symptomatology and MM outcomes.^48–50^^,^^60^ Two found that more lifetime psychotic or mood episodes were associated with MM in schizophrenia and bipolar disorder.^49^^,^^60^ One study found that a history of attempted suicide was associated with more comorbid medical conditions in schizophrenia.^50^ In bipolar disorder, those who experienced rapid cycling, depressive symptoms, comorbid anxiety disorders, and sleep disturbances, but not manic symptoms, reported significantly more medical disorders, though these effect sizes were weak.^49^ Another study reported no association between MM and comorbid mental disorders.^58^ SMI illness severity was not associated with MM risk in one study.^48^ No studies investigated the impact of negative symptoms (e.g. avolition) on MM risk.

#### Other Clinical Factors

Five studies reported contrasting results for the impact of psychiatric hospital admissions on MM outcomes.^9^^,^^48^^,^^50^^,^^52^^,^^60^ Three studies found that having a history of, or a higher number of psychiatric hospitalisations, was associated with increased MM risk.^9^^,^^48^^,^^60^ The remaining two studies did not observe such association.^50^^,^^52^ No significant associations between intellectual or physical disabilities and MM were observed.^9^^,^^58^

#### Pharmacological Treatment

Across three studies, the impact of antipsychotic medication and other pharmacologic treatments on MM was inconclusive.^48^^,^^49^^,^^59^ One study found treatment adherence to pharmacological therapies (e.g. first-generation antipsychotics) was associated with a greater number of comorbidities in schizophrenia,^59^ although another study did not observe any significant associations between first- or second-generation antipsychotics and MM^48^. Individuals with bipolar disorder who received second-generation antipsychotic treatment reported a significantly lower mean number of medical disorders than those who did not.^49^ There was no evidence for antidepressants, benzodiazepines, or mood stabilisers impacting MM outcomes.^48^^,^^49^ However, those who were prescribed anxiolytic/hypnotic medication reported a greater number of comorbidities, while those who were prescribed lithium reported significantly fewer comorbidities.^49^

Two studies investigated the impact of polypharmacy. One study found polypharmacy increased MM risk among schizophrenia and bipolar disorder groups,^60^ whereas another reported no significant differences in the number of medical comorbidities among individuals with bipolar disorder prescribed varying numbers of psychotropic medications.^49^

#### Health and Lifestyle Factors

Health and lifestyle factors for MM were investigated in five studies.^49^^,^^50^^,^^56^^,^^58^^,^^59^ Factors associated with increased MM risk included increased weight and waist circumference,^49^^,^^59^ being underweight (among men experiencing homelessness),^58^ physical inactivity,^56^ excessive caloric intake,^56^ and consumption of psychoactive substances,^59^ though two studies did not observe a significant association between substance abuse and MM outcomes.^50^^,^^56^ Four studies reported mixed findings on the impact of smoking.^49^^,^^56^^,^^58^^,^^59^ One study found daily tobacco smokers had fewer comorbidities than non-smokers,^49^ whereas another observed a positive relationship between the number of cigarettes smoked and the number of comorbidities, though statistics were not reported for this finding.^59^ Two studies found no association between smoking and MM.^56^^,^^58^ No significant associations between excessive alcohol intake and MM were found.^50^^,^^56^^,^^58^

### Conceptual Framework

**Figure 2:**
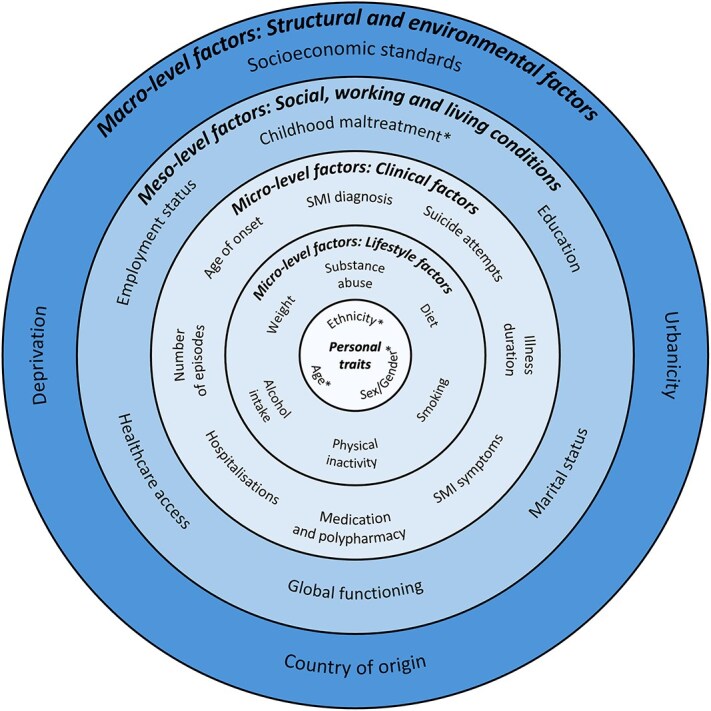
Visual Representation of the Conceptual Framework for the Factors Associated With Physical Multimorbidity in Severe Mental Illness (SMI). Asterisks (^*^) indicate demographic or psychosocial factors with the strongest evidence.

Figure 2 presents a conceptual framework to illustrate the complex landscape of factors associated with MM in SMI that were identified in this review. The demographic, psychosocial, clinical, and lifestyle factors synthesised here can be organised thematically into personal traits, micro-, meso-, and macro-levels. These levels encompass factors surrounding the self, clinical and disorder-specific factors, lifestyle factors, social, living and working conditions, and broader structural and environmental factors.

## Discussion

To our knowledge, this is the first systematic review concerned with psychosocial and demographic risk factors related to physical MM in SMI. Our key findings were that female gender/sex, older age, childhood maltreatment, and ethnicity were most strongly associated with MM. This systematic review builds on previous meta-analyses^4^^,^^13^ which establish robust prevalence estimates of MM in SMI by exploring psychosocial and demographic risk factors to help build a comprehensive model of risk.

### Factors Identified

The findings of this systematic review demonstrate the similarity between the risk factors associated with MM in the general and SMI populations. Research in the wider population provides support for older age,^67–69^ urbanicity,^70^ lower educational attainment,^71^ unemployment,^72^^,^^73^ and lifestyle factors (e.g. physical inactivity^74^) increasing MM risk. In the general population, those from ethnically minoritized (e.g. Indian and Black Caribbean) groups have been shown to have higher odds of cardiovascular MM relative to White British groups, independent of social-economic factors.^75^

However, our results suggest that the impact of these factors on MM risk is greater in SMI than the general population. For example, MM research in the general population has observed increased odds of up to 1.61 for Black ethnic groups,^75^^,^^76^ whereas SMI studies in this review report OR of up to 2.09.^25^ The impact of these factors on MM may therefore be magnified in SMI and operate via different vulnerability pathways (e.g. clinical factors) to influence disease onset. Similarly, older age is associated with MM in SMI^2^^,^^25^^,^^40^^,^^49^^,^^50^^,^^53^^,^^55–57^^,^^59^^,^^63^^,^^65^ and the general population.^67–69^ However, older age is generally defined as being over 40 or 50 in this review on SMI, but over 65 years in general population studies.^77^ The lower age threshold suggests that SMI groups may be susceptible to accelerated ageing and higher rates of age-related physical conditions.^78^

Moreover, childhood maltreatment was consistently associated with MM in bipolar disorder.^39^^,^^49^^,^^51^^,^^65^ Although this pattern is consistent with what is observed in the general population,^79^ the underlying mechanism or pathway may differ since childhood maltreatment is linked to a worse course of illness in bipolar disorder^80^^,^^81^ resulting in polypharmacy, potentially increasing the risk of physical conditions due to its adverse side effects (e.g. insulin resistance^82^). However such pathways are yet to be formally tested.

It is possible that MM manifests differently in those with SMI and can be explained through a syndemic framework,^83^ whereby interactions between psychosocial and clinical factors exacerbate physical health burden. Syndemic theory emphasises the importance of social context when understanding the co-occurrence of health conditions, and that macro- and micro-level factors should be considered in combination.^83^ However, most studies in this review investigated the individual rather interactional impact of risk factors on MM outcomes. Future SMI research would benefit from adopting a more holistic, systems-level approach to MM in a syndemic context to better inform the development of effective MM interventions in SMI.^83^

The proposed conceptual framework integrates SMI-specific factors and highlights themes consistent with those outlined in existing public health models in the general population (e.g. Krieger’s Ecosocial Theory^84^ and Dahlgren and Whitehead’s Model of Health Determinants^85^), suggesting that MM is driven by a multitude of systemic factors at personal, micro-, meso-, and macro-levels. Thematically, the identified psychosocial and demographic factors suggest that social disadvantage is associated with MM. For example, those from minoritised ethnic groups, residing in deprived areas, and a history of childhood maltreatment were associated with elevated MM risk relative to advantaged counterparts.

### Directions for Future Research

The disparate evidence prohibits the establishment of these factors as definitive determinants of MM in SMI. Given that MM rates are significantly higher in SMI relative to the general population,^4^ it is likely that other factors driving MM risk have been missed in the limited research conducted so far (e.g. social support). This stresses the importance of lived experience research to uncover factors for further investigation and contextualize current findings (e.g. how marital status contributes to MM). Given that few studies investigated interactions between factors, consideration of the impact multiple disadvantage or intersectionality is not possible but is worthy of future investigation.^86^ Nonetheless, the conceptual framework can act as a useful tool to highlight vulnerability factors for MM in SMI and aid further investigation.

The findings illustrate a complex landscape of psychosocial and demographic risk factors for MM in SMI. However, an understanding of the mechanisms involved is notably lacking. Authors across the included studies similarly propose that stress, allostatic load, and inflammation may underlie the associations between psychosocial factors and MM,^25^^,^^39^^,^^49^^,^^87^ though biological pathways have not yet been examined in this context. Similarly, gene-environment interplay in the development of MM is underexplored. Research indicates genetic predispositions to physical comorbidities in SMI,^88^^,^^89^ and findings in the general population suggest that social determinants of health may heighten susceptibility to adverse health outcomes through epigenomic mechanisms.^90^ Furthermore, neurobiological alterations underlying SMI, such as immunosuppression^91^ and metabolic syndrome^92^ may elevate the risk of MM independently of psychosocial influences, warranting acknowledgement in MM’s etiology. Future research focused on biological (e.g. inflammatory biomarkers, genetics), psychological (e.g. intrinsic motivation), and societal mechanisms (e.g. violence against women) promises to advance knowledge in this area, potentially identifying clinical targets.

### Clinical Implications

The findings shed light on high-risk or vulnerable groups (i.e. female gender/sex, older age, childhood maltreatment, and ethnically minoritised groups) who would benefit most from prevention and intervention investment. For example, existing interventions such as increasing culturally informed physical health education for people from minority ethnic backgrounds and healthcare access for those in deprived areas, can be tailored to individuals with SMI. The outcomes of such work would include reduction in personal, societal and economic burdens.

### Limitations and Strengths

There are several methodological strengths of this study which include a systematic search, quality appraisal, and comprehensive synthesis of the available literature on psychosocial and demographic factors linked to MM in SMI for the first time. But limitations of this systematic review and included studies must be considered when interpreting the findings. In terms of the included studies there are 3 main limitations to highlight. Firstly, heterogeneous MM definitions were employed across studies which precluded a meta-analysis and reduce the ability to draw valid conclusions. It is crucial that a universal MM definition is adopted by future studies to generate more conclusive evidence about its determinants to inform effective intervention development. Drawing on the Academy of Medical Sciences definition^93^ we recommend that physical MM in SMI be operationalised as: the coexistence of two or more long-term non-communicable or infectious *physical* conditions in an SMI population. Conceptualising MM in this way can promote holistic approaches to health management, rather than viewing each additional physical health condition as a separate comorbidity.^7^

Secondly, different SMI diagnoses were considered across studies, which may partly explain the mixed findings. For example, different SMI diagnoses have different psychopharmacological interventions,^94^^,^^95^ which may introduce different MM outcomes. Future studies should therefore stratify their findings by SMI diagnoses. Moreover, childhood maltreatment was the most consistently reported risk factor for MM across the studies, but all studies focused on bipolar disorder. Future investigations should extend this work to other SMI diagnoses.

Thirdly, statistical relationships between factors could not be considered for several studies since they did not use inferential statistics or provide the necessary information for the effects to be calculated.^45^^,^^64^ Although we included these studies to ensure comprehensive synthesis of the available literature, their findings should be treated cautiously. For methodological reasons causality or direction of associations could not be inferred between risk factors and MM for the majority of included studies. This is crucial to establish so that such research can accurately inform prevention and intervention efforts. For instance, unemployment might lead to higher MM rates, but MM may reduce employment opportunities leading to unemployment. Future studies should use study designs (e.g. longitudinal cohorts) and statistical approaches that allow for causality or direction of associations to be determined.

There are four methodological limitations that may introduce risk of bias, thus our findings should be interpreted cautiously. First, included studies were limited to those published in English potentially excluding studies published in other languages. Second, the search strategy may have missed studies that did not conceptualise psychosocial or demographic factors of MM as “risk factors”, “determinants”, “correlates”, or “predictors”. Thirdly, clinical and lifestyle factors were not systematically searched for, so the included literature is unlikely to reflect all of the available research, but are considered in other studies.^12–14^ For example, in this review included studies did not examine negative symptoms (e.g. avolition), although they can lead to physical comorbidities in SMI^96^ since they are linked to poor self-care.^97–99^ Healthcare fragmentation was not captured in included studies, but may also contribute to MM^100^, by limiting individuals with SMI from seeking timely care. To advance the field future studies need to investigate the synergistic impact of clinical, lifestyle and psychosocial risk factors to build a comprehensive model of MM risk for this population.

## Conclusion

This systematic review is the first to synthesize the literature on the psychosocial and demographic factors associated with MM in SMI. We found that female gender/sex, older age, childhood maltreatment, and ethnicity were most strongly associated with MM in SMI. These may highlight groups at increased risk of MM and may benefit most from prevention and intervention efforts. However, this area remains understudied, and the findings for other factors were more mixed. Future research should adopt a consistent MM definition and investigate interactions between these factors to build a holistic risk/aetiological model.

## Supplementary Material

R_and_R_Supplementary_material_sbaf128
