# Supplementary material for: Psychosocial and Demographic Factors Associated with Physical Multimorbidity in Severe Mental Illness: A Systematic Review
Source: Schizophr Bull. 2025 Aug 28;52(4):sbaf128. doi: 10.1093/schbul/sbaf128 (PMC13391624; doi:10.1093/schbul/sbaf128)

**Supplementary Material**

Table of Contents

[Table of Contents 1](#__RefHeading___Toc200125262)

[Supplementary Table 1. Eligibility criteria for papers to be included in this review 2](#__RefHeading___Toc200125263)

[Supplementary Table 2. Factors associated with physical MM with effect sizes. 3](#__RefHeading___Toc200125264)

[Supplementary Table 3. Key findings of studies that did not report inferential statistics. 14](#__RefHeading___Toc200125265)

[Supplementary Table 4. Clinical factors associated with multimorbidity in the included studies 20](#__RefHeading___Toc200125266)

[Supplementary Table 5. Lifestyle factors associated with multimorbidity in the included studies 22](#__RefHeading___Toc200125267)

[Supplementary Table 6. Quality appraisal of included studies using Joanna Briggs Institute (JBI) quality appraisal checklists 23](#__RefHeading___Toc200125268)

[Supplementary Table 7. Physical health conditions studied across included studies 25](#__RefHeading___Toc200125269)

[Supplementary Table 8. Search strategy 36](#__RefHeading___Toc200125270)

[Supplementary Table 9. PRISMA checklist 37](#__RefHeading___Toc200125271)

[Supplementary Table 10. Completed ROBIS tool 39](#__RefHeading___Toc200125272)

Supplementary Table 1. Eligibility criteria for papers to be included in this review

| **Framework** | **Inclusion** | **Exclusion** |
| --- | --- | --- |
| Population | Severe mental illness (SMI):   - Must study individuals with SMI - Includes non-affective and affective psychotic disorders (e.g. ICD-10 codes F20-F29) and bipolar disorder (F31) - Studies that looked at individuals with psychotic features were considered. | The sample includes participants with SMI but is not stratified to show findings specific to people with SMI |
| Exposure | Demographic and psychosocial risk factors:   - Must look at the association between at least one demographic or psychosocial factor and physical multimorbidity | Demographic and psychosocial risk factors are not investigated as predictors or associates of physical multimorbidity |
| Outcome | Physical multimorbidity:   - Must be the outcome in the study - Defined as two or more physical health conditions in addition to SMI diagnosis - Studies can also investigate the total number of comorbid physical health conditions as the outcome | Physical multimorbidity is not measured as the outcome |

Supplementary Table 2. Factors associated with physical MM with effect sizes.

| **Study** | **Factors associated with MM** | **Findings** | |
| --- | --- | --- | --- |
|  |  | Significant | Non-significant |
| Bouza et al. (2010) | Female gender | Mean number of ICD-9 codes (SD):  Women 1.26 (1.46)  Men 1.20 (1.34)  Women had significantly more ICD-9 codes than men; *p* = .002, Hedges’ g (95% CI) = 0.04 (0.01 – 0.08). | - |
| de Freitas et al. (2022) | Ethnicity  Gender  Older age  Area-level deprivation | Adjusted odds ratios (AORs) and 95% confidence intervals (CI) for having MM, AOR (95% CI):  Ethnicity (compared to White British people):  *1 or more physical health conditions:*  Black British/Other Black background 1.64 (1.49 – 1.81)  Black African 1.41 (1.23 – 1.56)  Black Caribbean 1.79 (1.58 – 2.03)  Chinese 0.60 (0.43 – 0.88)  Other ethnic background 0.66 (0.58 – 0.75)  *3 or more physical health conditions:*  Black British 2.06 (1.83 – 2.31)  Black African 1.50 (1.33 – 1.70)  Black Caribbean 2.09 (1.81 – 2.42)  Other White background 0.78 (0.66 – 0.92)  Chinese 0.47 (0.27 – 0.81)  Other ethnic background 0.50 (0.41 – 0.61)  Gender (compared to men):  *3 or more physical health conditions:*  Women 0.90 (0.83 – 0.98)  Age (compared to 13-17 years):  *1 or more physical health conditions:*  50-65 years 1.53 (1.32 – 1.78)  *3 or more physical health conditions:*  50-65 years 1.60 (1.31 – 1.96)  Area-level deprivation (compared to least deprived [IMD deciles 7-10]):  *1 or more physical health conditions:*  Middle deprivation (IMD deciles 5-6) 1.26 (1.11 – 1.43)  Second most deprived (IMD deciles 3–4) 1.27 (1.13 – 1.43)  Most deprived (IMD deciles 1–2) 1.32 (1.18 – 1.49)  *3 or more physical health conditions:*  Middle deprivation (IMD deciles 5-6) 1.50 (1.25 – 1.79)  Second most deprived (IMD deciles 3–4) 1.56 (1.32 – 1.84)  Most deprived (IMD deciles 1–2) 1.61 (1.37 – 1.90) | Adjusted odds ratios for having MM, AOR (95% CI):  Ethnicity (compared to White British people):  *1 or more physical health conditions:*  Other White background 0.95 (0.84 – 1.07)  Asian British/Other Asian background 1.05 (0.90 – 1.23)  Irish 0.94 (0.74 – 1.21)  Indian 1.25 (0.97 – 1.60)  Pakistani 1.03 (0.76 – 1.38)  Bangladeshi 0.96 (0.66 – 1.42)  Mixed ethnic background 1.15 (0.97 – 1.36)  *3 or more physical health conditions:*  Asian British/Other Asian background 1.06 (0.87 – 1.30)  Irish 1.02 (0.75 – 1.39)  Indian 1.26 (0.92 – 1.71)  Pakistani 0.89 (0.59 – 1.33)  Bangladeshi 0.78 (0.46 – 1.33)  Mixed ethnic background 1.22 (0.98 – 1.52)  Gender (compared to men):  *1 or more physical health conditions:*  Women 0.97 (0.92 – 1.04)  Age (compared to 13-57 years):  *1 or more physical health conditions:*  18-34 years 1.05 (0.91 – 1.21)  18-34 years 1.13 (0.99 – 1.31)  *3 or more physical health conditions:*  18-34 years 1.15 (0.96 – 1.39)  18-34 years 1.14 (0.95 – 1.38)  The effects of ethnicity on multimorbidity did not differ by gender or level of deprivation as indicated by the statistically non-significant interaction terms in the logistic models [likelihood ratio (LR) test based chi-squared = 14.45(12), *p* = .273 for ethnicity × gender and 44.48(36), *p* = .148 for ethnicity × deprivation interactions respectively]. |
| Dixon et al. (1999) | Female gender  Older age  Being married  Recruitment from public healthcare (relative to private)  History of attempted suicide | Older individuals, women, persons who were married, and persons recruited to the sample from public institutions reported more current medical problems.  Older age: r = .31; df = 717, p < .005  T-tests for the mean number of comorbid medical conditions between groups, Mean (SD):  Gender: Male 0.68 (0.98)  Female 0.94 (1.04)  *t*(717) = -3.39, *p* < .005  Marital status:  Ever married 0.97 (0.90)  Never married 0.58 (1.09)  *t*(715) = -5.23, *p* < .005  Healthcare status:  Private 0.45 (0.79)  Public 0.82 (1.03)  *t*(717) = -3.35, *p* < .005  Age:  < 40 years 0.48 (0.77)  > 40 years 1.02 (1.11)  *t*(717) = -7.38, *p* < .005  Ever actually attempted suicide:  Yes 0.88 (1.02)  No 0.70 (0.99)  *t*(713) = -2.31, *p* = .02  *Note:* Authors were contacted to provide further information to calculate effect sizes  Multivariate analyses:  In multivariate analysis, marital status was excluded because of its high correlation with age causing a problem with multicollinearity. Although women were older than men in this sample, there was no evidence that this association caused a problem with multicollinearity. Thus, gender was retained as an independent variable in the analysis. Only older age (beta = .26, p < .005) emerged as a significant predictor of numbers of current medical problems in the analysis. The F-value obtained was significant (F = 9.09, df = 703, p < .005). | Bivariate analyses, Mean (SD):  Race:  White 0.75 (0.97)  African American/Asian/Other 0.80 (1.06)  *t*(714)= -0.60, *p* = .55  Site:  Rural 0.80 (1.03)  Urban 0.77 (1.01)  *t*(717) = 0.32, *p* = .75  US State:  Southern 0.73 (0.99)  Midwestern 0.80 (1.02)  *t*(717)= -0.99, *p* = .32  Hospital setting:  Community 0.82 (1.00)  Hospital 0.70 (1.02)  *t*(717)= 1.57, *p* = .12  Alcohol/drug disorder:  No 0.79 (1.03)  Yes 0.73 (0.97)  *t*(717) = 0.85, *p* = .39  Education:  < 12 years 0.79 (1.01)  > 12 years 0.73 (1.01)  *t*(717) = 0.82, *p* = .41 |
| Fenn et al. (2005) | Older age | The prevalence of both current and lifetime medical comorbidity increases with age (respectively: F(5) = 19.8, p < .001; F(5) = 18.1,  *p* < .001).  *Note:* Authors were contacted to provide further information to calculate effect sizes | - |
| Filipcic et al. (2019) | Female gender  Low educational level  Number of hospitalisations | Women had more than double the odds of having chronic physical MM than men (OR = 2.47; 95% CI 1.35 – 4.50), while the gender-related burden of chronic diseases in controls was nearly the same (OR = 0.89; 95% CI 0.65 – 1.22).  The combination of age and gender indicated that the highest burden was seen in younger ages (<35 years), with a strong overrepresentation of women. Women SSD patients <35 years old had a significantly higher prevalence of CPM than women control participants (X^2^ = 9.8, *p* = .002; false discovery rate < 5%). A significant difference between men SSD patients and control participants <35 years old was not observed (X^2^ = 1.7, *p* = .194).  Multivariable binary logistic regressions on prevalence of chronic physical multimorbidity, OR (95% CI):  Gender (relative to Men):  Women 2.47 (1.35 – 4.50), *p =* .003  Education (relative to Primary level):  University 0.37 (0.15 – 0.95), *p =* .039  Number of hospitalisations:  1.05 (1.00 – 1.10), *p* = .041 | Multivariable binary logistic regressions on prevalence of chronic physical multimorbidity, OR (95% CI):  Education (relative to Primary level):  Secondary 0.84 (0.39-1.84), *p =* 0.666  Age (years):  1.00 (0.97 – 1.03), *p =* .972  Work status (relative to Employed):  Unemployed 1.63 (0.82 – 3.24), *p =* .162  Retired 2.02 (1.00 – 4.24), *p =* .062  Marital status (relative to Single):  Married 1.71 (0.77 – 3.77), *p =* .186  Widowed or divorced 0.76 (0.32 – 1.81), *p =* .536  Diagnosis (relative to Schizophrenia [F20]):  Acute and transient Psychotic disorder [F23] 0.69 (0.29 – 1.65),  *p* = .402  Schizoaffective disorder [F25] 0.49 (0.21 – 1.12), *p* = .090  Unspecified nonorganic Psychosis [F29] 0.60 (0.26 – 1.39), *p* = .230  Persistent delusional Disorder [F22] 1.34 (0.37 – 4.90), *p* = .656  Schizotypal disorder [F21] 2.00 (0.34 – 11.71), *p =* .441  Duration of illness (years):  1.03 (0.99 – 1.06), *p* = .142  Clinical global impression severity scale severity (CGI-S, relative to Up to mildly ill):  Moderately ill 0.45 (018 – 1.13), *p* = .089  Significantly ill 0.44 (0.17 – 1.14), *p* = .092  Severely ill 0.53 (0.18 – 1.57),  *p* = .255  Treatment with antipsychotics:  First generation 1.72 (0.83 – 3.55), *p* = .146  Second generation 1.09 (0.60 – 1.98), *p* = .774  Antidepressants:  0.67 (0.37 – 1.22), *p* = .188  Benzodiazepines:  1.85 (0.92 – 3.71), *p* = .085 |
| García-Goñi  et al.  (2021) | Socioeconomic inequality | The overall negative concentration indices revealed that, after controlling for age and sex, people residing in more deprived areas presented more chronic conditions compared to those who lived in more affluent areas. The only positive index was the one corresponding to no chronic conditions at all, meaning that better health was more concentrated among the rich.  Age and sex-adjusted concentration index of socioeconomic inequality, Concentration Index (SE):  1 condition: 0.044 (6.026), *p* < .05  9 conditions: -0.084 (-2.165), *p* < .05  10 or more conditions: -0.177 (-5.175), *p* < .05 | Age and sex-adjusted concentration index of socioeconomic inequality, Concentration Index (SE):  2 conditions: 0.006 (0.542), *p* > .10  3 conditions: -0.008 (-0.636), *p* > .10  4 conditions: -0.015 (-1.081), *p* > .10  5 conditions: -0.036 (-1.961), *p* < .10  6 conditions: -0.046 (-2.023), *p* < .10  7 conditions: -0.056 (-2.029), *p* < .10  8 conditions: 0.001 (0.020), *p* > .10  *Note:* It was inferred that *p* > .10 for concentration indexes when the authors did not specify whether *p* was < .05 or < .10 in the paper. |
| Godin et al. (2023) | Sex (female)  Older age  Not having a Bachelor degree  Longer bipolar illness duration (> 15 years)  Number of bipolar lifetime episode (tertile)  Bipolar subtype  Rapid cycling  Depressive symptoms  Anxiety disorder  Non-tobacco smokers  Sleep disturbances (PSQI)  BMI above 25  No current 2nd generation antipsychotic prescription  No current lithium prescription  Anxiolytic/hypnotic medication  Childhood maltreatment (CTQ total)  Childhood emotional neglect  Childhood emotional abuse  Childhood abuse (sum of abuse and neglect)  Childhood sexual abuse | Medical morbidity by levels of exposure to childhood maltreatment, Kruskal-Wallis or Mann-Whitney tests, Number of medical comorbidities mean (SD):  CTQ total score:  1^st^ tertile 1.47 (1.45)  2^nd^ tertile 1.66 (1.53)  3^rd^ tertile 1.81 (1.66)  *p* < .001  Emotional neglect:  No (no/low) 1.57 (1.51)  Yes (moderate/severe) 1.82 (1.65)  *p* < .001  Emotional abuse:  No (no/low) 1.56 (1.47)  Yes (moderate/severe) 1.87 (1.73)  *p* < .001  Sexual abuse:  No (no/low) 1.59 (1.50)  Yes (moderate/severe) 1.93 (1.74)  *p* < .001  Univariate associations between variables at baseline and the number of medical disorders, Medical morbidity mean (SD):  Sex:  Female 1.7 (1.6)  Male 1.5 (1.5)  *p* < .001; Hedges g (95% CI) = 0.13 (0.06 – 0.21)  Age:  <40 (median) 1.5 (1.5)  >40 1.8 (1.6)  *p* < .001; Hedges g (95% CI) = -0.19 (-0.26 – -0.11)  High school (Bachelor degree):  No 1.8 (1.6)  Yes 1.6 (1.6)  *p* = .006; Hedges g (95% CI) = 0.12 (0.04 – 0.21)    BD illness duration:  <15 years (median) 1.5 (1.5)  >15 years 1.8 (1.6)  *p* < .001; Hedges g (95% CI) = -0.19 (-0.26 – -0.11)  Number of lifetime mood episodes (tertile):  <4 episodes 1.5 (1.4)  4-8 episodes 1.6 (1.5)  >8 episodes 1.9 (1.7)  *p* < .001; <4 vs. >8 episodes: Hedges g (95% CI) = -0.24 (-0.34 – -0.13)  BD subtype:  Type I 1.6 (1.6)  Type II 1.7 (1.6)  Type NOS 1.7 (1.5)  *p* = .02; Type I vs Type II: Hedges g (95% CI) = -0.06 (-0.14 – 0.01)  Rapid cycling:  No 1.6 (1.5)  Yes 1.9 (1.7)  *p* = .009; Hedges g (95% CI) = -0.18 (-0.28 – -0.07)  Depressive symptoms (MADRS):  <=8 1.5 (1.4)  >8 1.8 (1.6)  *p* < .001; Hedges g (95% CI) = -0.19 (-0.26 – -0.11)  Anxiety disorders:  No 1.5 (1.5)  Yes 1.8 (1.6)  *p* < .001; Hedges g (95% CI) = -0.19 (-0.26 – -0.11)  Current daily tobacco smoking:  No 1.7 (1.6)  Yes 1.6 (1.5)  *p* = .02; Hedges g (95% CI) = 0.07 (-0.01 – 0.14)  Sleep disturbances (PSQI):  No 1.4 (1.4)  Yes 1.8 (1.6)  *p* < .001; Hedges g (95% CI) = -0.25 (-0.33 – -0.17)  BMI > 25:  No 1.5 (1.5)  Yes 1.8 (1.6)  *p* < .001; Hedges g (95% CI) = -0.19 (-0.26 – -0.11)  Second generation antipsychotics:  No 1.8 (1.6)  Yes 1.6 (1.5)  *p* = .02; Hedges g (95% CI) = 0.13 (0.05 – 0.22)  Lithium:  No 1.8 (1.6)  Yes 1.6 (1.5)  *p* = .02; Hedges g (95% CI) = 0.13 (0.04 – 0.22)  Anxiolytic/hypnotics:  No 1.6 (1.5)  Yes 1.9 (1.7)  *p* = .004; Hedges g (95% CI) = -0.18 (-0.27 – -0.08)  CTQ total score (tertile):  1^st^ tertile 1.47 (1.45)  2^nd^ tertile 1.66 (1.53)  3^rd^ tertile 1.81 (1.66)  *p* < .001  Emotional neglect:  No 1.57 (1.51)  Yes 1.82 (1.65)  *p* < .001  Emotional abuse:  No 1.56 (1.47)  1.87 (1.73)  *p* < .001  Sexual abuse:  No 1.59 (1.50)  Yes 1.93 (1.74)  *p* < .001  Linear regression (multivariate associations adjusted for potential confounding of sex, age, BMI, PSQI, Anxiety disorders and density of episodes):  Observed associations between medical morbidity, childhood maltreatment (based on tertiles of CTQ total score), sum of childhood abuse and the presence of sexual abuse (respectively *p* = .008, *p* < .001 and *p* = .03).  Standardised coefficients:  CTQ score tertiles: Beta = .071; *t* = 2.657; *p* =.008  Childhood abuse (sum of abuse and neglect): Beta = .09; *t* = 3.344; *p* < .001  Sexual abuse: Beta = .057; *t* = 2.18; *p* = .029  Sex, age, BMI, PSQI, anxiety disorders and density of episodes were all shown to be significant predictors of medical morbidity when included in the respective models at *p* < .05 | Medical morbidity by levels of exposure to childhood maltreatment, Kruskal-Wallis or Mann-Whitney tests, Number of medical comorbidities mean (SD):  Physical neglect:  No (no/low) 1.63 (1.53)  Yes (moderate/severe) 1.76 (1.69)  *p* = .27  Physical abuse:  No (no/low) 1.62 (1.52)  Yes (moderate/severe) 1.85 (1.76)  *p* = .10  Univariate associations (Medical morbidity mean [SD]):  Age at BD onset:  <21 years 1.6 (1.5)  >21 years 1.7 (1.6)  *p* = .89  Manic symptoms (YMRS):  <=8 1.6 (1.5)  >8 1.7 (1.7)  *p* = .99  Substance abuse:  No 1.6 (1.5)  Yes 1.7 (1.6)  *p* = .22  Number pf psychotropic medications:  1 medication 1.6 (1.5)  2-3 medications 1.7 (1.6)  >3 medications 1.8 (1.6)  *p* = .14 |
| Hosang et al. (2018) | Childhood maltreatment (abuse and neglect)  Childhood abuse  Childhood neglect | Interaction effects of mood disorder status and childhood maltreatment on diagnoses of having a number of medical illnesses, adjusted for gender and age, AOR (95% CI):  Bipolar disorder × childhood maltreatment 7.18 (2.23 – 23.08), *p* = .001  Bipolar disorder × abuse 8.70 (2.49 – 30.33), *p* = .001  Bipolar disorder × neglect 3.58 (1.01 – 12.73), *p* = .049  Dose-response relationship:  For the bipolar group the odds for being diagnosed with at least one or a greater number of medical illnesses was highest among those who had been exposed to two or more types of maltreatment (number of medical disorders: AOR = 5.13, 95% CI 1.42 – 18.50, *p* = .012), followed by the those who were subject to one form (number of medical illnesses: AOR = 4.26, 95% CI 1.31 – 13.84, *p* = .016) compared with those without a history of childhood maltreatment.  When the results were adjusted for duration of illness for mood disorder and age of mood disorder onset, a graded relationship between number of maltreatment histories and medical morbidity was no longer observed, whereby the odds of having a medical illness was similar for those who reported one or two or more forms of maltreatment. |  |
| Jahrami et al. (2017) | Older age  Excessive caloric intake  Inadequate physical activity (inactivity) | Association between lifestyle factors and medical comorbidities, OR (95% CI):  2 comorbidities:  Age: 1.10 (1.05 – 1.14), *p* = .001  Excessive caloric intake: 4.95 (1.65 – 14.8), *p* = .004  ≥ 3 comorbidities:  Age: OR 1.19 (1.10 – 1.28), *p* = .001  Inadequate physical activity (inactivity): 8.67 (1.22 – 61.41), *p* = .03  Excessive caloric intake: 4.47 (1.10 – 18.14), *p* = .036 | Association between lifestyle factors and medical comorbidities, OR (95% CI):  2 comorbidities:  Sex: 2.99 (0.91-9.87), *p* = .07  Smoking: 2.94 (0.87-9.86), *p* = .08  Alcohol intake: 1.53 (0.27-8.50), *p* = .62  Inadequate physical activity (inactivity): 1.52 (0.49 – 4.60),  *p* = .46  ≥ 3 comorbidities:  Sex: 2.98 (0.69 – 12.78), *p* = .14  Smoking: 1.07 (0.25 – 4.45), *p* = .92  Alcohol intake: 2.70 (0.20 – 35.47), *p* = .44 |
| Lasebikan & Azegbeobor (2017) | Poorer functioning  Longer duration of illness  Higher number of episodes  Polypharmacy  Hospitalisation | Binary regression of clinical features of patients with multiple medical comorbidity (≥3 general medical conditions) adjusted for age and gender, AOR (95% CI):  *Schizophrenia group:*  Duration of illness (relative to <2 years):  ≥ 2 years 1.90 (1.42 – 2.58), *p* < .001  ≥ 3 episodes (relative to <3 episodes): 1.59 (1.22 – 2.05), *p* < .001  Polypharmacy (relative to no polypharmacy): 1.46 (1.13 – 1.81), *p* = .003  Global Assessment of Functioning score (relative to ≥ median):  < Median 1.40 (1.06 – 1.74), *p* = .01  Even been hospital (relative to never hospitalised) 1.61 (1.19 – 2.10), *p* <.001  *Bipolar group:*  Duration of illness (relative to <2 years):  ≥ 2 years 1.43 (1.09 – 1.89), *p* = .02  ≥ 3 episodes (relative to <3 episodes): 1.59 (1.29 – 2.12), *p* < .001  Polypharmacy (relative to no polypharmacy): 1.32 (1.00 – 1.74), *p* < .05  Global Assessment of Functioning score (relative to ≥ median):  < Median 1.52 (1.18 – 2.00), *p* < .001  Even been hospital (relative to never hospitalised): 1.49 (1.13 – 1.99), *p* < .002 | - |
| Mirza et al. (2021) | Earlier age at diagnosis  SMI diagnosis (schizophrenia spectrum disorder, relative to bipolar affective disorder)  Hospitalisations | Chi-square tests for differences between individuals without complex multimorbidity  vs individuals with complex multimorbidity:  Age at diagnosis:  15-24: 37.9% vs 62.1%  25-34: 36.5% vs 63.5%  35-44: 38.4% vs 61.6%  45-54: 37.3% vs 62.7%  55-64 38.7% vs 61.3%  65-74: 42.5% vs 57.5%  75+: 46.5% vs 53.5%  *X^2^* = 39.95 (6), *p* < .001  OR (95% CI):  75+ (relative to 15-24): 0.70 (0.60 – 0.82), *p* < .01  SMI diagnosis:  Schizophrenia spectrum disorder: 37.5% vs 62.5%  Bipolar affective disorder: 41.7% vs 58.3%  *X^2^* = 19.47 (1), *p* < .001  OR (95% CI):  SSD (vs. Bipolar affective disorder): 1.20 (1.10 – 1.29);  *p* < .01  Hospitalisations:  Yes: 35.5% vs 64.5%  No: 42.1% vs 57.9%  *X^2^* = 63.317 (1), *p* < .001  OR (95% CI):  Hospitalisations (vs. no hospitalisations): 1.32 (1.23, 1.41), *p* < .01 | Chi-square tests for differences between individuals without complex multimorbidity  vs individuals with complex multimorbidity:  Sex:  Male: 38.1% vs 61.9%  Female: 38.9% vs 61.1%  *X^2^* = 0.98 (1), *p* = .322  Ethnicity:  British White 39.2% vs 60.8%  Black African 36.7% vs 63.3%  Black Caribbean 39.0% vs 61.0%  South Asian 41.9% vs 58.1%  Irish White 37.9% vs 62.1%  Chinese 37.0% vs 63.0%  Unknown 38.2% vs 61.8%  *X^2^* = 5.95 (5), *p* = .312  IMD:  1 (most deprived) 40.8% vs 59.2%  2 37.8% vs 62.2%  3 39.4% vs 60.6%  4 38.5% vs 61.5%  5 (least deprived) 38.0% vs 62.0%  Unknown 35.1% vs 64.9%  *X^2^* = 2.21 (4), *p* = .697  Intellectual disabilities:  F7: Mild intellectual disabilities 34.0% vs 66.0%  *X^2^* = 2.29 (1), *p* = .130  F8: Developmental disorders 35.6% vs 64.4%  *X^2^* = 0.830 (1), *p* = .362 |
| Post et al. (2013) | For having 4+ medical comorbidities (as compared to 1-3):  Higher total childhood adversity score (tCAS)  Being from the US (as opposed to Europe [The Netherlands and Germany])  Female gender  For having 1-3 medical comorbidities (as compared to none):  Higher total childhood adversity score  Increased age  Female gender | Multinomial logistic regression, RRR (95% CI):  Significant independent predictors of having 4+ medical comorbidities as compared to 1–3 included higher tCAS score (the best predictor), being from the US, and female gender, but not age.  Significant predictors of having 1–3 medical comorbidities as compared to none included higher tCAS score, increased age, and female gender, but not country of origin.  *4+ comorbidities (relative to 1-3):*  Total Childhood Adversity Score: 1.26 (0.17 – 0.29), *p* < .01  European country (relative to US): 0.58 (-1.06 – -0.03), *p* = .04  Male gender (relative to female): 0.66(-0.82 – -0.01), *p* = .05  *No comorbidities (relative to 1-3):*  Total Childhood Adversity Score: 0.92 (-0.14 – -0.02), *p* = .01  Age: 0.98 (-0.03 – 0.00), *p* = .01  Male gender (relative to female): 1.66 (0.18 – 0.84), *p <* .01 | Multinomial logistic regression, RRR (95% CI):  *4+ comorbidities (relative to 1-3):*  Age: 1.01 (-0.01 – 0.03), *p* = .25  *No comorbidities (relative to 1-3):*  European country (relative to US): 1.21 (-0.16 – 0.54), *p* = .28 |
| Public Health England (2018) | Older age | Prevalence of having 3 or more physical health comorbidities in SMI patients, % (95% CI):  Age group:  15-34 years: 0.44 (0.23 – 0.83)  35-54 years: 2.99 (2.52 – 3.54)  55-74 years: 11.17 (10.10 – 12.34)  *Note:* Authors were contacted to provide further information to calculate effect sizes  When compared to non-SMI patients:  Compared to all patients, younger patients with SMI diagnosed with 3 or more physical health conditions show the highest level of inequality. Patients with SMI are 5 times more likely to have 3 or more physical health conditions whereas for ages 55 to 74 this is 1.4 higher. Although rate ratio in the prevalence of multi-morbidities patients aged 15 to 34 is great it must be remembered that overall the prevalence is low. | Prevalence of having 3 or more physical health comorbidities in SMI patients, % (95% CI):  Sex:  Female: 4.03 (3.52 – 4.60)  Male: 4.67 (4.10 – 5.30)  Townsend deprivation quintile:  5 (least deprived): 3.83 (2.82 – 5.07)  4: 3.27 (2.33 – 4.45)  3: 4.97 (3.99 – 6.11)  2: 4.08 (3.32 – 4.97)  1 (most deprived): 4.30 (3.48 – 5.26) |
| Rodrigues et al. (2022) | Material deprivation  Persistence of impaired functioning (need for social assistance) | Prevalence ratios (PR) of sociodemographic and clinical factors and their association with multimorbidity (2+ conditions) among people with psychotic disorders at 10-year follow-up, PR (95% CI):  Gradient effect for neighbourhood-level material deprivation, with people living in the two most deprived areas having a greater prevalence of multimorbidity (Second Highest Quintile: PR = 2.46, 95% CI 0.99 – 6.12; Highest Quintile: PR = 3.09, 95% CI 1.21 – 7.90).  Material deprivation (relative to Quintile 1 [least deprived]):  Quintile 2 1.69 (0.57 – 5.07)  Quintile 3 2.14 (0.76 – 6.05)  Quintile 4 2.46 (0.99 – 6.12)  Quintile 5 (most deprived) 3.09 (1.21 – 7.90)  Persistence:  Length of time on Ontario Drug Benefit (ODB) (365-day intervals) 1.06 (1.00 – 1.12)  The prevalence of multimorbidity among people with psychotic disorders increased by 5% for each 30-day increase in total hospital days (PR = 1.05, 95% CI 0.96 – 1.13), although this included the possibility of a null effect. The prevalence of 2 + chronic conditions among people with psychotic disorders increased by 6% or each year of social assistance through ODB (PR = 1.06, 95% CI 1.00 – 1.12). | Prevalence ratios (PR) of sociodemographic and clinical factors and their association with multimorbidity (2+ conditions) among people with psychotic disorders at 10-year follow-up, PR (95% CI):  Age group at baseline/first episode of psychosis (relative to 16-20 years):  21-25 years 1.33 (0.72-2.46)  26-30 years 1.77 (0.98-3.21)  People aged 26–30 years at the first episode of psychosis had a 77% higher prevalence of multimorbidity at 10-year follow-up (PR = 1.77, 95% CI 0.98–3.21), relative to people aged 16–20 years, although our 95% CI included the null value.  Sex (relative to males):  Females 1.01 (0.59-1.73)  Severity:  Total hospital days (30-day intervals) 1.05 (0.96-1.13) |
| Rojanaworarit et al. (2025) | Underweight BMI | Multivariable binomial generalized linear regression models with identity link functions, prevalence difference (PD), 95% CI:  The multivariable model revealed that the only significant factor related to the increased prevalence of multimorbidity was underweight after adjusting for other covariates. Underweight individuals had a 15% significantly greater prevalence of multimorbidity compared to individuals with normal weight.  Underweight BMI (relative to normal weight):  0.150 (0.017–0.823), *p* = .027 | Univariable binomial generalized linear regression models with identity  link functions, prevalence difference (PD), 95% CI:  From the univariable analysis, positive values of prevalence difference (PD) indicating greater prevalence of multimorbidity were identified in the groups of individuals who were underweight, overweight, ever or current drinkers, having physical disabilities, and having co-occurring mental disorders. However, none of these positive PD values were statistically significant.  Age > 40 years (relative to < 40 years):  -0.014 (-0.161, 0.132), *p* = .847  BMI (relative to normal weight):  Underweight 0.128 (-0.006, 0.261), *p* = .060  Overweight or obesity 0.013 (-0.211, 0.238), *p* = .908  Ever or current smoker (relative to non-smoker):  -0.053 (-0.201, 0.094), *p* = .480  Ever or current alcohol drinker (relative to non-drinker):  0.069 (-0.060, 0.198), *p* = 0.295  Physical disabilities (relative to no disabilities):  0.108 (-0.170, 0.387), *p* = 0.446  Co-occurring mental disorders (relative to none):  0.150 (-0.120-0.419), *p* = 0.276  Multivariable binomial generalized linear regression models with identity  link functions, prevalence difference (PD), 95% CI:  Although factors including age ≥40 years, overweight or obese, being ever or current alcohol drinker, having physical disabilities, and having co-occurring mental disorders also showed positive values of adjusted PD; none of these factors obtained statistically significant results  Age > 40 years (relative to < 40 years):  0.019 (-0.128, 0.166), *p* = .803  BMI (relative to normal weight):  Overweight or obesity 0.034 (-0.190, 0.256), *p* = .760  Ever or current smoker (relative to non-smoker):  -0.091 (-0.242, 0.060), *p* = .239  Ever or current alcohol drinker (relative to non-drinker):  0.108 (-0.026, 0.241), *p* = 0.113  Physical disabilities (relative to no disabilities):  0.046 (-0.237, 0.328), *p* = 0.752  Co-occurring mental disorders (relative to none):  0.181 (-0.091-0.454), *p* = 0.191 |
| Smith et al. (2013a) | Female gender | Women with schizophrenia were significantly more likely to have two or more physical conditions (18.1% vs 12.9%; *p* < .001) and three or more physical conditions than men (23.9% vs 12.6%; *p* < .001).  Women with schizophrenia were significantly more likely to have 2 or more physical comorbidities relative to men (OR 2.13, 95% CI 1.95-2.32, *p* < .01). | - |
| Smith et al. (2013b) | Female gender | Women with bipolar disorder were significantly more likely to have three or more physical conditions than men (25.0% versus 17.0%; *p* < .001).  Women with bipolar disorder were significantly more likely to have 2 or more physical comorbidities than men (OR 2.20, 95% CI 1.87 – 2.59, *p* < .01). | This gender difference was not significant for two physical comorbidities (17.4% women versus 15.1% men; *p* = .10) |
| Stapp et al. (2020) | Childhood sexual abuse  Childhood emotional abuse | Association of each type of childhood maltreatment with medical morbidity 3 years later, IRR (95% CI):  Sexual abuse and emotional abuse were significantly associated with greater medical morbidity three years later in mania/hypomania.  Sexual abuse: 1.08 (1.04 – 1.11), *p* < .001  Emotional abuse: 1.05 (1.01 – 1.10), *p* = .017 | Association of each type of childhood maltreatment with medical morbidity 3 years later, IRR (95% CI):  Physical abuse: 1.01 (0.98 – 1.04), *p* = .46  Physical neglect: 1.02 (0.99 – 1.05), *p* = .16  Emotional neglect: 1.01 (0.98 – 1.05), *p* = .43 |
| Stubbs et al. (2016) | Younger age | Number of physical health conditions across psychosis groups, Mean (SD):  Subclinical psychosis 0.83 (0.99)  Psychosis diagnosis 1.27 (1.33)  Difference in mean number of physical health conditions between those with subclinical psychosis versus psychosis diagnosis is significant at *p* < .0001, Hedges’ g (95% CI) = 0.44 (0.40 – 0.49) | - |
| Teh et al. (2021) | Older age  Earlier age of psychotic illness onset  Ethnicity (Malay and Indian ethnic groups as compared to Chinese ethnic group) | Chi-square analyses:  As compared to other non-affective and affective psychotic disorders, schizophrenia was significantly related to a greater frequency of comorbid physical conditions (X^2^ = 6.63 and X^2^ = 4.07 respectively, *p* < .05).  Frequency of having two or more comorbid conditions, *n* (%):  Schizophrenia 29 (8)  Non-affective psychotic disorders 9 (2.5)  Affective psychotic disorders 2 (0.6)  Multinomial regression analyses of having Two comorbidities vs None, OR (95% CI):  Older age, earlier age of onset, Malay and Indian ethnic groups (as compared to Chinese ethnic group) were risk factors associated with greater odds of having one, and two or more comorbid conditions as compared to none.  Age:  1.12 (1.076 – 1.168), *p* < .001  Age of onset:  0.94 (0.891 – 0.989), *p* = .018  Ethnicity (relative to Chinese):  Malay 3.41 (1.248 – 9.336), *p* = .017  Indian 3.32 (1.134 – 9.701), *p* = .029 | Multinomial regression analyses of having Two comorbidities vs None, OR (95% CI):  Marital status, education levels, and the type of psychotic diagnosis were not significantly associated with comorbidity.  Ethnicity (relative to Chinese):  Others 0.77 (0.084 – 7.095), *p* = .820  Sex (relative to Male):  Female 0.61 (0.285 – 1.305), *p* = .202  Marital status (relative to being single):  In a relationship/married 1.20 (0.457 – 3.086), *p* = .724  Divorced/separated/widowed: 0.76 (0.165 – 3.532), *p* = .731  Education (relative to Secondary and below):  Pre-tertiary 1.73 (0.669 – 4.468), *p* = .259  Tertiary and above 1.11 (0.423 – 2.903), *p* = .835  Psychiatric diagnoses (relative to schizophrenia):  Other non-affective psychotic disorders 1.01 (0.421 – 2.437), *p* = .977  Affective psychotic disorders 1.40 (0.230 – 8.502), *p* = .716 |
| Thabet et al. (2019) | Gender  Older age  Unemployment  Being married  Urbanicity  Consumption of psychoactive substances  Waist circumference  Drug adherence | Bivariate associations:  Age:  The older the patient, the greater the number of somatic comorbidities (*p* = .001; *r* = 0.36).  Gender:  The male subjects were more likely to develop somatic comorbidities (100% versus 88.9%, *p* = .051). The male gender correlated with a larger number of comorbidities (4.13 versus 2.83, *p* = .007). Hedges’ g (95% CI) = 0.74 (0.21– 1.27)  Marital status:  Being married correlated with a greater number of these comorbidities (5 versus 3.2, *p* < .01). Hedges’ g (95% CI) = 1.05 (0.56– 1.55)  Urbanicity:  Living in urban areas correlated with a greater number of comorbidities (4.2 versus 3.1, *p* = .02)  Hedges’ g (95% CI) = 0.62 (0.16– 1.08)  Employment status:  The unemployed individuals with schizophrenia had more comorbidities than their professionally active counterparts (4.5 versus 3.6, *p* = .05). Hedges’ g (95% CI) = 0.42 (0.10– 0.93)  Weight:  The number of somatic comorbidities positively correlated with weight and waist circumference respectively *p* = .016; *r* = 0.272 and *p* = .005; *r* = 0.318).  Psychoactive substances:  Psychoactive substances consumption correlated with a significant number of somatic comorbidities. People who took psychoactive substances reported more comorbidities than those who did not (4.30 versus 2.52, *p* = .000).  Hedges’ g (95% CI) = 0.92 (0.41– 1.42)  Multiple linear regression with number of comorbidities as the dependent variable, Beta Coefficient:  Age 0.391, *p* = .002  Consumption of psychoactive substances 0.509, *p* < .001  Waist circumference 0.241, *p* = .010  Drug adherence 0.205, *p* = .026 | Bivariate associations:  Socioeconomic standards:  There was no statistically significant relationship between low socioeconomic standards and somatic comorbidities (*p* = .264 and .701 respectively).  Education:  Low educational level did not correlate significantly with comorbidities (*p* = .51 and .402, respectively).  Smoking:  The greater the number of cigarettes consumed per year, the more important the number of comorbidities.  *Note:* Authors were contacted to provide further information of this effect size.  Adherence to therapeutic follow-up:  A poor adherence to a therapeutic follow-up did not correlate with the prevalence of somatic comorbidities (*p* = 1.00).  Concerning the combination therapy (APs), it was associated with a greater number of somatic comorbidities (*p* = .061; *r* = 0.213). |

Supplementary Table 3. Key findings of studies that did not report inferential statistics.

| **Study** | **Factors associated with MM** | **Effect sizes reported in paper** | **Calculated statistical test** |
| --- | --- | --- | --- |
| Bouza et al. (2010) | Older age | The number of ICD-9 codes increased with age. For patients aged 53 years or more, 84% had at least one physical ICD-9 code (17% had one code; 24% two codes; and 43% three or more codes). This increase with age occurred for both men and women. | *Note:* Authors were contacted to provide further information to calculate effect sizes |
| Charlson et al. (2020) | Ethnicity | Number of comorbid diagnoses, Mean (SE):  Ethnicity:  Aboriginal: 1.0 (0.07)  Torres Strait Islander: 0.40 (0.06)  Sex:  Male: 0.7 (0.06)  Female: 0.8 (0.09)  Frequency of two comorbid physical disorders:  Ethnicity:  Aboriginal: 45 (17.4%)  Torres Strait Islander 8 (6.1%)  Sex:  Male: 38 (13.6%)  Female: 18 (12.3%)  Frequency of three or more comorbid physical disorders:  Ethnicity:  Aboriginal: 28 (10.8%)  Torres Strait Islander 4 (3.0%)  Sex:  Male: 20 (7.1%)  Female: 12 (8.2%) | Ethnicity:  Aboriginal people had significantly higher rates of MM relative to Torres Strait Islander people (OR = 3.84, 95% CI 3.07 – 7.75; *p* < .01).  *X^2^* = 17.375 (1); *p* < .01  Sex:  There were no significant sex differences in MM outcomes.  *X^2^* = 4.185e-31 (1); *p* = 1.00 |
| Domino et al. (2014) | Female sex  Ethnicity | Sex (MM vs no MM):  Male 34.5% vs 65.5%  Female 46.1% vs 53.9%  Ethnicity (MM vs no MM):  Unspecified 41.5% vs 58.5%  African American 40.1% vs 59.9%  Latino 34.2% vs. 65.8% | Sex:  Females had significantly higher rates of MM relative to males (OR = 1.62, 95% CI 1.52 – 1.73, *p* < .01).  *X^2^* = 216.54 (1), *p* < .01  Ethnicity:  Latino people with schizophrenia had significantly lower rates of MM relative to those whose ethnicity was unspecified (OR = 0.73, 95% CI 0.57 – 0.93, *p* < .01).  There were no significant differences in MM rates between African American individuals and those whose ethnicity was unspecified. |
| Gabilondo et al. (2017) | Female gender | Mean number of chronic physical illnesses was 1.37 for women with schizophrenia and 1.04 for men with schizophrenia  Frequency of having 2 chronic physical illnesses among people with schizophrenia (%):  Women 16.8  Men 12.6  Frequency of having 3 or more chronic physical illnesses among people with: schizophrenia:  Women 18.4  Men 12.9 | Women had significantly higher rates of MM (2 or more physical illnesses) relative to men (OR = 1.59, 95% CI 1.44-1.77, *p* < .01).  *X^2^* = 80.71 (1), *p* < .01 |
| Hsu et al. (2021) | Male sex  Older age | Among elderly patients with late-life schizophrenia, males reported a higher Charlson Comorbidity Index (CCI) score relative to females:  Mean CCI score (SD):  Male 2.04 (2.09)  Female 1.59 (1.83)  Patients aged 85+ reported higher CCI scores relative to those aged between 65-74 and 75-84 years:  Mean CCI score (SD):  65-74: 1.52 (0.79)  75-84: 2.25 (2.14)  85+: 2.96 (2.31) | Sex:  Welch Modified Two-Sample T-Test:  Male elderly patients with late-life schizophrenia reported a significantly higher CCI score relative to females, *t*(3820) = 7.10, *p* < .01, Hedges’ g (95% CI) = 0.25 (0.18 – 0.31).  Age group:  *65-74 vs 75-84:*  Welch Modified Two-Sample T-Test:  Patients aged between 65-74 reported a significantly lower CCI score relatively to those aged between 75-84 years, *t*(1319) = -11.29, *p* < .01, Hedges' g (95% CI) = 0.92 (0.85 – 1.00).  *65-74 vs 85+:*  Welch Modified Two-Sample t-Test:  Patients aged between 65-74 reported a significantly lower CCI score relatively to those aged 85+, *t*(232) = -9.36, *p* < .01, Hedges’ g (95% CI) = 1.82 (1.68 – 1.97).  *75-84 vs. 85+:*  Standard two-sample t-test:  Patients aged between 75-84 reported a significantly lower CCI score relative to those aged 85+, *t*(1392) = -4.52, *p* < .01, Hedges’ g (95% CI) = 0.33 (0.19 – 0.47).  *Note:* Bonferroni-adjusted *p*-value to account for multiple comparisons between age groups was *p* = .02 |
| Launders et al. (2022) | Female sex  Older age  SMI diagnosis | Multimorbidity was more common in females and in older age groups.  MM more common in females among younger age groups:  Age 18-29: males 5.78% versus females 9.94%  Age 30-39: males 9.46% vs females 16.83%  Age 40-49: males 19.95%; females 27.56%  MM more common in older age across both genders:  Males at 80+ 79.65%; females 76.49%  MM (more than one physical health condition) was more common in Other and Bipolar disorder SMI diagnoses  Having more than one physical health condition:  Schizophrenia: 28.49%  Bipolar: 35.30%  Other: 35.73% | Sex:  Across all SMI diagnoses (and age groups), females had a significantly higher prevalence of MM than males.  *X^2^* = 1547.7 (1), *p* < .01  OR = 1.90, 95% CI 1.84-1.96, *p* < .01.  Age:  Across all SMI diagnoses, individuals aged 60+ years had significantly higher prevalence rates of MM than those aged 18-59 years.  *X^2^* = 13667 (1), *p* < .01  OR = 7.90, 95% CI 7.61-8.19, *p* < .01  SMI diagnosis:  Individuals with schizophrenia had significantly lower rates of MM compared to individuals with bipolar disorder or other SMI diagnoses.  *X^2^* = 260.36 (2), *p* < .01  OR (95% CI) relative to bipolar disorder:  Schizophrenia 0.73 (0.70 – 0.76), *p* < .01  Other SMI diagnoses 1.02 (0.98 – 1.06), *p* = 0.30 |
| Mirabzadeh et al. (2020) | Older age | MM was more prevalent among older patients with schizophrenia.  Frequency of comorbid non-psychiatric disorders among schizophrenia Patients ≤65 years vs. ≥65 years:  No disorder 91.67% vs. 21.37%  1 disorder 5.30% vs. 18.32%  2 disorders 3.03% vs. 22.90%  3 disorders 0 vs 21.38%  4 disorders 0 vs 16.03% | Age:  Patients with schizophrenia aged 65 years and above had significantly higher rates of MM relative to those below 65 years of age.  *X^2^* =163.92 (1), *p* < .01  OR = 47.16, 95% CI 22.65 – 111.80, *p* < .01 |
| Monk et al. (2024) | Ethnicity | Māori individuals with psychosis had a higher mean M3 score relative to non-Māori individuals  M3 mean (SD):  Māori 0.22 (0.48)  Non-Māori 0.19 (0.45) | Welch Modified Two-Sample T-Test:  Māori individuals with psychosis had a significantly higher mean M3 score relative to non-Māori individuals, *t*(13944) = 4.41, *p* < .01, Hedges’ g (95% CI) = 0.07 (0.04 – 0.10). |
| Owen et al. (2023) | High area-level deprivation | P = psychosis, D = diabetes, C = congestive heart failure  Area-level deprivation:  For all combinations of what order the illnesses started, the prevalence rates were highest in the most deprived quintiles than the least  Most deprived vs least deprived quintile  PDC - 36.2% vs 7.6%  PCD - 27.0% vs 18.3%  DPC - 29.0% vs 11.1%  DCP - 29.1% vs 13.1%  CPD - 28.8% vs 17.5%  CDP - 26.7% vs 18.1%  Sex:  Female vs male  PDC - 51.1% vs 48.9%  PCD - 59.1% vs 40.9%  DPC - 54.4% vs 45.6%  DCP - 54.4% vs 45.6%  CPD - 48.8% vs 51.3%  CDP – 52.6% vs 47.4% | Area-level deprivation:  A significant difference in MM rates between the most deprived and least deprived areas was observed, whereby people in the most deprived quintile had significantly higher rates of MM relative to the least deprived quintile.  *X^2^* = 6.42 (1), *p* = .01  OR = 1.28, 95% CI 1.06 – 1.54, *p* < .01  Sex:  No significant sex differences in MM outcomes were observed.  *X^2^* = 0.25 (1), *p* = .62 |
| Reilly et al. (2015) | SMI diagnosis (Unspecified/other affective psychosis)  Deprivation (only for bipolar disorder and unspecified/other affective psychosis) | Mean (SD) number of comorbidities across SMI diagnoses:  Schizophrenia: 0.8 (1.2)  Bipolar: 1.0 (1.3)  Unspecified/Other affective psychosis: 1.2 (1.4)  Other types of psychosis: 0.9 (1.3)  Mean (SD) number of comorbidities in the most deprived vs. most affluent quintiles, by SMI diagnoses:  All SMI diagnoses: 1.0 (1.3) vs 1.0 (1.3)  Schizophrenia: 0.8 (1.2) vs 0.9 (1.4)  Bipolar: 1.1 (1.3) vs 1.0 (1.3)  Unspecified/Other affective psychosis: 1.3 (1.4) vs 1.1 (1.4)  Other types of psychosis: 0.9 (1.3) vs 0.9 (1.2) | SMI diagnoses:  Individuals with unspecified/other affective psychosis reported a significantly higher mean number of comorbidities relative to other SMI diagnoses.  *Schizophrenia vs. Bipolar disorder:*  Individuals with schizophrenia reported a significantly fewer comorbidities relative to those with bipolar disorder, *t*(15747)= -10.22, *p* < .008,  Hedges’ g (95% CI) = 0.15 (0.12 – 0.18).  *Schizophrenia vs. Unspecified/other affective psychosis:*  Individuals with schizophrenia reported fewer comorbidities relative to those with unspecified/other affective psychosis, *t*(6368)= -14.62, *p* < .008,  Hedges’ g (95% CI) = -0.29 (-0.33 – -0.25).  *Schizophrenia vs. Other types of psychosis:*  Individuals with schizophrenia reported fewer comorbidities relative to those with other types of psychosis, *t*(15746)= -5.34, *p* < .008, Hedges’ g (95% CI) = -0.08 (-0.11 – -0.05).  *Bipolar disorder vs. Unspecified/other affective psychosis:*  Individuals with bipolar disorder reported significantly fewer comorbidities relative to those with unspecified/other affective psychosis, *t*(6102)= -7.44, *p* < .008,  Hedges’ g (95% CI)= -0.14 (-0.18 – -0.10).    *Bipolar disorder vs. Other types of psychosis:*  Individuals with bipolar disorder reported significantly more comorbidities relative to those with unspecified/other affective psychosis, *t*(21125)= 5.56, *p* < .008,  Hedges’ g (95% CI) = 0.08 (0.05 – 0.10).  *Unspecified/other affective psychosis vs Other types of psychosis:*  Individuals with unspecified/other affective psychosis reported significantly more comorbidities relative to those with other types of psychosis, *t*(5662)= 11.43, *p* < .008, Hedges’ g (95% CI) = 0.23 (0.19 – 0.27).  *Note:* Bonferroni-adjusted *p*-value to account for multiple comparisons between age groups was *p* = .008  Most deprived versus most affluent quintiles, by SMI diagnosis:  *All SMI diagnoses:*  There were no significant differences in the mean number of comorbidities between individuals with SMI residing in the most deprived versus the most affluent quintiles, *t*(11763)= 0.00, *p* = 1.00.  *Schizophrenia:*  There were no significant differences in the mean number of comorbidities between individuals with schizophrenia residing in the most deprived versus the most affluent quintiles,  *t*(1474)= -1.80, *p* = .07.  *Bipolar disorder:*  Individuals with bipolar disorder residing in the most deprived quintile reported significantly more comorbidities than those residing in the most affluent quintile, *t*(3454)= 2.26, *p* = .02, Hedges’ g (95% CI) = 0.08 (0.01 – 0.14).  *Unspecified/other affective psychosis:*  Individuals with unspecified/other affective psychosis residing in the most deprived quintile reported significantly more comorbidities than those residing in the most affluent quintile, *t*(1255) = 2.52, *p* = .01, Hedges’ g (95% CI) = 0.14 (0.03 – 0.25).  *Other types of psychosis:*  There were no significant differences in the mean number of comorbidities between individuals with other types of psychosis residing in the most deprived versus the most affluent quintiles,  *t*(4005) = 0.00, *p* = 1.00. |
| Stubbs et al. (2016) | Younger age | Association between psychosis and multimorbidity (outcome) estimated by multivariable logistic regression analysis, OR (95% CI):  Multimorbidity was most evident in younger age groups, with people aged 18–44 years with psychosis at greatest odds of physical health multimorbidity:  Subclinical psychosis (vs controls):  18-44 years 2.71 (2.40-3.05), *p* < .001  45-64 years 1.93 (1.68-2.20), *p* < .001  ≥ 65 years 1.69 (1.40-2.04), *p* < .001  Psychosis diagnosis (vs controls)  18-44 years 4.68 (3.46-6.32), *p* < .001  45-64 years 3.78 (2.77-5.16), *p* < .001  ≥ 65 years 2.22 (1.34-3.68), *p* < .01  Odds for multimorbidity in low-income and middle-income countries were similar:  Subclinical psychosis (vs controls):  Low-income countries 2.03 (1.80 – 2.28)  Middle-income countries 2.43 (2.16 – 2.74)  Psychosis diagnosis (vs controls):  Low-income countries 3.99 (3.00 – 5.31)  Middle-income countries 4.07 (2.93 – 5.65) | *Note:* Authors were contacted to provide further information to calculate effect sizes |

Supplementary Table 4. Clinical factors associated with multimorbidity in the included studies

| **Study** | **Earlier age of SMI illness onset** | **Longer duration of SMI illness** | **Number of SMI episodes** | **Hospitalisations** | **Attempted suicide** | **Polypharmacy** | **Antipsychotic treatment** | **Other medication** | **Intellectual disabilities** | **Illness severity** | **Rapid cycling** | **Depressive symptoms** | **Manic symptoms** | **Anxiety disorders** | **SMI diagnosis** | **Sleep disturbances** | **Physical disabilities** |
| --- | --- | --- | --- | --- | --- | --- | --- | --- | --- | --- | --- | --- | --- | --- | --- | --- | --- |
| Dixon et al.  (1999) | - | - | - | **X** Hospital treatment setting (vs. community) | **↑** | - | - | - | - | - | - | - | - | - | - | - | - |
| Filipcic et al. (2019) | - | **X**^◊^ | - | **↑** Number of hospitalisations^◊^ | - | - | **X** First generation antipsychotics^◊^  **X** Second generation antipsychotics^◊^ | **X** Antidepressants^◊^  **X** Benzodiazepines^◊^ | - | **X**^◊^ | - | - | - | - | **X** SSD diagnoses^◊^ | - | - |
| Godin et al. (2023) | **X** | **↑** | **↑** Lifetime episodes  **↑** Rapid cycling | - | - | **X** | **↓** Second generation antipsychotics | **X** Antidepressants  **X** Mood stabilisers    **↓** Lithium  **↑** Anxiolytic/ hypnotics | **-** | - | **↑** | **↑** | **X** | **↑** | **↑** Bipolar disorder subtypes | **↑** | **-** |
| Lasebikan & Azegbeobor  (2017) | - | **↑** | **↑** | **↑** | - | **↑** | - | - | **-** | - | - | - | - | - | - | - | - |
| Launders et al. (2022) | - | - | - | - | - | - | - | - | - | - | - | - | - | - | **↓** Schizophrenia (vs. bipolar disorder or other SMI diagnoses)† | - | - |
| Mirza et al.  (2021) | **↑** | - | - | **↑** | **-** | - | - | - | **X** | **-** | - | **-** | - | - | **↑** Schizophrenia (vs. bipolar disorder) | - | - |
| Reilly et al. (2015) | - | - | - | - | - | - | - | - | - | - | - | - | - | - | **↑** Unspecified/other affective psychosis (vs. other SMI diagnoses) |  | - |
| Rodrigues et al. (2022) | **X** | - | - | **X** Total hospital days | - | - | - | - | - | - | - | - | - | - | - | - | - |
| Rojanaworarit et al. (2025) | - | - | - | - | - | - | - | - | **-** | - | - | **X** Co-occurring mental disorders (including depression and anxiety)^◊^ | - | **X** Co-occurring mental disorders (including depression and anxiety)^◊^ | **-** | - | **X**^◊^ |
| Stubbs et al. (2016) | - | - | - | - | - | - | - | - | **-** | - | - | - | - | - | **↑** Psychosis diagnosis (vs. subclinical psychosis) | - | - |
| Teh et al. (2021) | **↑** | - | - | - | - | - | - | - | **-** | - | - | - | - | - | **↑** Schizophrenia (vs. non-affective and affective psychotic disorder groups)  **X** Association between type of psychotic diagnosis and MM | - | - |
| Thabet et al. (2019) | - | - | - | - | - | - | **↑** Treatment adherence | **-** | **-** | - | - | - | - | - | - | - | - |

↑ Positive association between factor and prevalence/odds/incidence of MM

↓ Inverse association between factor and prevalence/odds/incidence of MM

X No association between factor and physical MM

- Association not studied

^◊^ Association adjusted for other factors/covariates

† Statistical significance was calculated using information available in the paper

Supplementary Table 5. Lifestyle factors associated with multimorbidity in the included studies

| **Study** | **Tobacco smoking** | **Substance abuse** | **Excessive alcohol intake** | **Weight** | **Physical inactivity** | **Excessive caloric intake** |
| --- | --- | --- | --- | --- | --- | --- |
| Dixon et al.  (1999) | - | **X** | **X** | **-** | **-** | **-** |
| Godin et al. (2023) | **↓** | **X** | **-** | **↑** BMI > 25 | **-** | **-** |
| Jahrami et al. (2017) | **X** | - | **X** | **-** | **X** 2 comorbidities  **↑** 3+ comorbidities | **↑** 2 comorbidities  **↑** 3+ comorbidities |
| Rojanaworarit et al. (2025) | **X**^◊^ | - | **X**^◊^ | **↑** Underweight BMI^◊^  **X** Overweight/obesity BMI^◊^ | **-** | **-** |
| Thabet et al. (2019) | **↑** | **↑** Consumption of psychoactive substances | **-** | **↑** Weight  **↑** Waist circumference | **-** | **-** |

↑ Positive association between factor and prevalence/odds/incidence of MM

↓ Inverse association between factor and prevalence/odds/incidence of MM

X No association between factor and prevalence/odds/incidence of MM

- Association not studied

^◊^ Association adjusted for other factors/Covariate / potential confounders

† Statistical significance was calculated using information available in the paper

| **Study** | **Design** | **Criterion** | | | | | | | | | | | **Score** | **Rating** |
| --- | --- | --- | --- | --- | --- | --- | --- | --- | --- | --- | --- | --- | --- | --- |
|  |  | **Were the criteria for inclusion in the sample clearly defined?** | **Were the study subjects and the setting described in detail?** | **Was the exposure measured in a valid and reliable way?** | **Were objective, standard criteria used for measurement of the condition?** | **Were confounding factors identified?** | **Were strategies to deal with confounding factors stated?** | **Were the outcomes measured in a valid and reliable way?** | **Was appropriate statistical analysis used?** |  | | |  |  |
| Dixon et al. (1999) | Cross-sectional | 1 | 1 | 1 | 1 | 0 | 0 | 0 | 1 |  | | | 5 | Moderate |
| Fenn et al. (2005) |  | 1 | 0 | 1 | 1 | 0 | 0 | 1 | 1 |  | | | 5 | Moderate |
| Filipcic et al. (2019) |  | 1 | 1 | 1 | 0 | 1 | 1 | 1 | 1 |  | | | 7 | High |
| Gabilondo et al. (2017) |  | 1 | 1 | 1 | 1 | 1 | 1 | 1 | 1 |  | | | 8 | High |
| García-Goñi et al. (2021) |  | 1 | 1 | 1 | 1 | 1 | 1 | 1 | 1 |  | | | 8 | High |
| Godin et al. (2023) |  | 1 | 1 | 1 | 1 | 1 | 1 | 1 | 1 |  | | | 8 | High |
| Lasebikan & Azegbeobor (2017) |  | 1 | 1 | 1 | 1 | 1 | 1 | 1 | 1 |  | | | 8 | High |
| Mirabzadeh et al. (2020) |  | 1 | 1 | 1 | 1 | 0 | 0 | 1 | 0 |  | | | 5 | Moderate |
| Mirza et al. (2021) |  | 1 | 1 | 1 | 1 | 1 | 1 | 1 | 1 |  | | | 8 | High |
| Monk et al. (2024) |  | 1 | 1 | 1 | 1 | 1 | 1 | 1 | 1 |  | | | 8 | High |
| Post et al. (2013) |  | 1 | 0 | 1 | 1 | 1 | 1 | 0 | 1 |  | | | 6 | High |
| Public Health England (2018) |  | 1 | 1 | 1 | 1 | 0 | 0 | 1 | 1 |  | | | 6 | High |
| Rojanaworarit et al. (2025) |  | 1 | 1 | 1 | 1 | 1 | 1 | 1 | 1 |  | | | 8 | High |
| Smith et al. (2013a) |  | 1 | 1 | 1 | 1 | N/A | N/A | 1 | 1 |  | | | 6 | High |
| Smith et al. (2013b) |  | 1 | 1 | 1 | 1 | N/A | N/A | 1 | 1 |  | | | 6 | High |
| Stubbs et al. (2016) |  | 1 | 1 | 1 | 1 | 1 | 1 | 1 | 1 |  | | | 8 | High |
| Teh et al. (2021) |  | 1 | 1 | 1 | 1 | 1 | 1 | 1 | 1 |  | | | 8 | High |
| Thabet et al. (2019) |  | 1 | 1 | 0 | 1 | 0 | 0 | 1 | 0 |  | | | 4 | Moderate |
|  |  | **Were the two groups similar and recruited from the same population?** | **Were the exposures measured similarly to assign people to both exposed and unexposed groups?** | **Was the exposure measured in a valid and reliable way?** | **Were confounding factors identified?** | **Were strategies to deal with confounding factors stated?** | **Were the groups/participants free of the outcome at the start of the study (or at the moment of exposure)?** | **Were the outcomes measured in a valid and reliable way?** | **Was the follow up time reported and sufficient to be long enough for outcomes to occur?** | **Was follow up complete, and if not, were the reasons to loss to follow up described and explored?** | **Were strategies to address incomplete follow up utilized?** | **Was appropriate statistical analysis used?** |  |  |
| de Freitas et al. (2022) | Cohort | N/A | 1 | 1 | 1 | 1 | 1 | 1 | 1 | 1 | 1 | 1 | 10 | High |
| Domino et al. (2014) |  | 1 | 1 | 0 | 1 | 1 | 0 | 1 | 0 | 0 | N/A | 1 | 6 | Moderate |
| Hsu et al. (2021) |  | N/A | N/A | 1 | 1 | 1 | 1 | 1 | 1 | 0 | 0 | 1 | 7 | Moderate |
| Launders et al. (2022) |  | 1 | 1 | 1 | 1 | 1 | 1 | 1 | 1 | 1 | 1 | 1 | 11 | High |
| Owen et al. (2023) |  | N/A | 1 | 1 | 1 | 1 | 1 | 1 | 1 | 0 | 0 | 1 | 8 | Moderate |
| Reilly et al. (2015) |  | 1 | 1 | 1 | 0 | 0 | 0 | 1 | 1 | 1 | 0 | 1 | 7 | Moderate |
| Rodrigues et al. (2022) |  | 1 | 1 | 1 | 1 | 1 | 0 | 1 | 1 | 0 | 0 | 1 | 8 | Moderate |
| Stapp et al. (2020) |  | 1 | 1 | 1 | 1 | 1 | 0 | 0 | 0 | 0 | 0 | 1 | 6 | Moderate |
|  |  | **Were the groups comparable other than the presence of disease in cases or the absence of disease in controls?** | **Were cases and controls matched appropriately?** | **Were the same criteria used for identification of cases and controls?** | **Was exposure measured in a standard, valid and reliable way?** | **Was exposure measured in the same way for cases and controls?** | **Were confounding factors identified?** | **Were strategies to deal with confounding factors stated?** | **Were outcomes assessed in a standard, valid and reliable way for cases and controls?** | **Was the exposure period of interest long enough to be meaningful?** | **Was appropriate statistical analysis used?** |  |  |  |
| Hosang et al. (2018) | Case-control | 1 | 1 | 1 | 1 | 1 | 1 | 1 | 1 | 1 | 1 |  | 10 | High |
| Jahrami et al. (2017) |  | 1 | 1 | 1 | 1 | 1 | 1 | 1 | 1 | 1 | 1 |  | 10 | High |
|  |  | **Was the sample frame appropriate to address the target population?** | **Were study participants sampled in an appropriate way?** | **Was the sample size adequate?** | **Were the study subjects and the setting described in detail?** | **Was the data analysis conducted with sufficient coverage of the identified sample?** | **Were valid methods used for the identification of the condition?** | **Was the condition measured in a standard, reliable way for all participants?** | **Was there appropriate statistical analysis?** | **Was the response rate adequate, and if not, was the low response rate managed appropriately?** |  |  |  |  |
| Bouza et al. (2010) | Prevalence | 1 | 1 | 1 | 1 | 1 | 1 | 1 | 1 | N/A |  |  | 8 | High |
| Charlson et al. (2020) |  | 1 | 1 | 1 | 1 | 1 | 1 | 0 | 1 | N/A |  |  | 7 | High |

## **Supplementary Table 6.** Quality appraisal of included studies using Joanna Briggs Institute (JBI) quality appraisal checklists

*Note.* The following scoring parameters were implemented: Checklist for Analytical Cross-Sectional Studies (score out of 8; Low 0-2, Moderate 3-5, High 6-8); Checklist for Cohort Studies (score out of 11; Low 0-4, Moderate 5-8, High 9-11); Checklist for Case-Control Studies (score out of 10; Low 0-3, Moderate 4-7, High 8-10) and Checklist for Prevalence Studies (score out of 9; Low 0-3, Moderate 4-6, High 7-9).

Supplementary Table 7. Physical health conditions studied across included studies

| **Study** | **Physical health conditions studied** | | |
| --- | --- | --- | --- |
| Bouza et al. (2010) | Infectious diseases  Neoplasms  Endocrine diseases  Haematological diseases  Neurological diseases  Diseases of the circulatory system  Respiratory diseases  Diseases of the digestive system  Diseases of the genitourinary tract  Complications of pregnancy, childbirth, and the puerperium  diseases of the skin and subcutaneous tissue  Diseases of the musculoskeletal system and connective tissue  Injury and poisoning  Drug, alcohol and tobacco abuse/dependency | | |
| Charlson et al. (2021) | Non-communicable diseases:  Diabetes  Cardiovascular disease  Respiratory disease  Renal disease  Neurological disease  Communicable diseases (sexually transmitted infections [STIs])  Injuries (excluding self-harm) | | |
| de Freitas et al. (2022) | Asthma  Bronchitis  Diabetes  Hypertension  Low blood pressure  Overweight or obesity  Rheumatoid arthritis | | |
| Dixon et al. (1999) | High blood pressure  Diabetes  Sexually transmitted diseases (STDs)  Cancer  Breathing problems  Heart problems  Bowel problems  Hearing problems  Eyesight problems  Teeth problems  Skin problems  Seizures | | |
| Domino et al. (2014) | Asthma  Chronic obstructive pulmonary disease  Diabetes  Hypertension  Hyperlipidemia  Seizure disorder | | |
| Fenn et al. (2005) | Any autoimmune  Rheumatoid arthritis  Systemic lupus erythematosis (SLE)  Any cardio/cerebrovascular  Stroke  Coronary artery disease (CAD)  Procedure for CAD including CABG, stent, or other  Hypertension (HTN)  Cardiac arrythmias  Any dermatologic  Any endocrine  Diabetes mellitus type I  Diabetes mellitus type II  Hyperthyroidism  Hypothyroidism  Any eyes, ears, nose, throat (EENT) disease  Any gastrointestinal  Esophageal varices  Peptic ulcer disease  Alcohol gastritis  Pancreatisis, alcoholic  Any genitourinary  Benign prostatic hypertrophy  Incontinence  Prostate or genitourinary Cancer | | Any hematologic  Any hepatic  Hepatitis B  Hepatitis C  Cirrhosis, alcoholic  HIV/AIDS  AIDS  HIV+ without clinical AIDS  Dyslipidemias including hypercholesterolemia, hyperlipidemia, hypertriglyceridemia  Any musculoskeletal  Degenerative disc disease  Fractures  Osteoarthiritis  Any neurologic  Head trauma with loss of consciousness  Head trauma without loss of consciousness  Peripheral neuropathies  Seizure disorder  Tardive dyskinesia  Any pulmonary  Asthma  Chronic obstructive pulmonary disease (COPD) or emphysema  Sleep apnea  Any renal  Chronic renal insufficiency |
| Filipcic et al. (2019) | Asthma (allergic asthma included)  Chronic bronchitis, chronic obstructive pulmonary disease, and emphysema  Myocardial infarction (heart attack) or chronic consequences of myocardial infarction  Coronary heart disease or angina pectoris  High blood pressure (hypertension)  Cerebrovascular insult (cerebral haemorrhage, cerebral thrombosis) or chronic consequences of stroke  Arthrosis (arthritis excluded)  Low back disorder or other chronic back defects  Neck disorder or other chronic neck defects  Diabetes mellitus  Allergy (rhinitis, hay fever, eye inflammation, dermatitis, food allergy, or other allergies [allergic asthma excluded]  Liver cirrhosis  Urinary incontinence, problems in controlling the bladder  Kidney disease  Obesity | | |
| Gabilondo et al. (2017) | Parkinson  Dementia  Viral hepatitis  Chronic constipation  Human immunodeficiency virus Diabetes  Emphysema, chronic bronchitis, chronic obstructive pulmonary disease Chronic liver or pancreatic disease  Psoriasis or eczema  Hypothyroidism  Dyspepsia  Epilepsy (currently treated)  Heart failure  Disorders of the immune system  Paralysis or muscular dystrophy  Blindness & low vision  Cerebrovascular disease  Hematologic chronic disorders  Low back pain  Irritable bowel syndrome  Deafness  Chronic kidney disease  Glaucoma | Transplant status  Chronic heart disease, others  Chromosomal anomalies or inherited metabolic disorders  Bronchiectasis  Inflammatory bowel disease  Asthma (currently treated)  Multiple sclerosis  Hypertension  Ischemic heart disease  Cancer  Prostatic hypertrophy  Peripheral vascular disease  Peripheral neuropathy  Chronic sinusitis  Diverticular disease of intestine  Gout  Atrial fibrillation  Degenerative joint disease  Rheumatoid arthritis and autoimmune and connective tissue diseases Osteoporosis  Migraine | |
| García-Goñi et al. (2021) | Asthma  Chronic kidney disease  Diabetes  Chronic obstructive pulmonary disease  Heart failure  HIV  Migraine  Multiple sclerosis | | |
| Godin et al. (2023) | Neurological disorders (headache/migraine, multiple sclerosis, epilepsy, meningitis, stroke, head injury)  Cardiovascular disorders (hypertension, coronary disease, myocardial infarction, cardiac dysrhythmia)  Endocrine and metabolic disorders (diabetes, thyroid disorders, hypercholesterolemia, hypertriglyceridemia)  Urinary tract disorders (nephropathy, acute retention of urine)  Skin disorders (psoriasis, eczema, toxidermia/rash, acne)  Liver and digestive disorders (cirrhosis, hepatitis, peptic ulcer, inflammatory chronic intestinal diseases)  Allergic and auto-immune-inflammatory disorders (asthma, allergy, systemic lupus erythematosus, rheumatoid polyarthritis)  Cancer and chronic infectious disorders (HIV, chronic viral hepatitis),  Hypertension  Hyperglycemia  Hypercholesterolemia  Hypertriglyceridemia | | |
| Hosang et al. (2018) | Heart problems (stroke, angina and heart attack)  Asthma  Diabetes (type 1 and type 2)  Arthritis (osteoarthritis and rheumatoid arthritis)  Hypertension  Epilepsy or convulsions  Osteoporosis  Multiple sclerosis | | |
| Hsu et al. (2021) | 17 comorbid conditions:  Myocardial infarction  Congestive heart failure  Peripheral vascular disease  Cerebrovascular disease  Dementia  Chronic pulmonary disease  Rheumatic disease  Peptic ulcer disease  Mild liver disease  Diabetes without chronic complication  Diabetes with chronic complication  Hemiplegia or paraplegia  Renal disease  Any malignancy (including lymphoma and leukemia, but not including malignant neoplasm of skin)  Moderate or severe liver disease  Metastatic solid tumor  AIDS/HIV. | | |
| Jahrami et al. (2017) | Diabetes type 2  Hypertension  Cardiovascular problems (angina, myocardial infarction, stroke, heart  failure, cardiomyopathy, heart arrhythmia, carditis, and venous  thrombosis)  Musculoskeletal disorders (osteoarthritis, gout,  rheumatoid arthritis, fibromyalgia, plantar fasciitis, heel spurs, and tendonitis) | | |
| Lasebikan & Azegbeobor (2017) | Nutritional anaemia  Hypertension  Ischaemic heart disease  Bronchial asthma  Chronic dermatitis  Diabetes mellitus  Thyrotoxicosis  Hypothyroidism  Chronic pain  Chronic headache  Peptic ulcer disease  Osteoarthritis haemorrhoids Malignancy  Common chronic infections such as tuberculosis, schistosomiasis, dracontiasis, malaria, hepatitis | | |
| Launders et al. (2022) | Asthma  COPD  Cardiac arrhythmia  Congestive heart failure  Myocardial infarction  Cerebrovascular disease  Neurological disorders (including epilepsy, multiple sclerosis, Parkinson disease, and seizures, but excluding cerebrovascular disease and dementia)  Cancer  Diabetes (type 1 or 2)  Hypothyroidism  Liver disease  Renal disease | Peptic ulcers  Rheumatic and collagen disease  Paresis or paralysis  HIV/AIDS  Hypertension  Peripheral vascular disease  Pulmonary circulation disorders  Valvular disease  Deficiency anaemia  Blood loss anaemia  Coagulopathy  Fluid or electrolyte disorders | |
| Mirabzadeh et al. (2020) | Musculoskeletal disorders  Cardiovascular disorders  Metabolic disorders  Hematological disorders  Endocrine disorders  Digestive disorders  Neurological disorders  Respiratory disorders  Dermatological disorders | | |
| Mirza et al. (2021) | Neoplastic disorders  Haematological disorders  Endocrine disorders  Neurological disorders  Eye and adnexal disorders  Circulatory disorders  Respiratory disorders  Digestive disorders  Dermatological disorder  Musculoskeletal disorders  Genitourinary disorders | | |
| Monk et al. (2024) | 50 physical health categories from the M3 multimorbidity index:  Cardiac arrhythmia  Cardiac disease other  Diabetes complicated  Hypertension uncomplicated  Metabolic disorder  Bowel disease inflammatory  Chronic pulmonary  Coagulopathy and other blood disorders  Myocardial infarction  Cerebrovascular disease  Angina  Congestive heart failure  Eye problem long term  Intestinal disorder  Chronic renal  Anemia deficiency  Obesity  Paralysis  Joint or spinal disorder  Cardiac valve  GI ulcer or upper GI disease  Inner ear disorder  Other neurological disorders*  Prostate cancer  Hepatitis Chronic viral  Metastatic cancer  Peripheral vascular disease  Endocrine disorder | | Diabetes uncomplicated  Dementia  Sleep disorder  Urinary tract problem (chronic)  Other cancers  Malignant melanoma  Breast cancer  Malnutrition nutritional  Pulmonary circulation disorders  Epilepsy  Connective tissue disease  Liver disease: moderate or severe  Pancreatitis  Colorectal cancer  Muscular peripheral nerve disorders  Lymphomas and leukaemias  Aortic and other aneurysms  Mental retardation  Bone disorders  Gynaecological cancers  Venous insufficiency  Osteoporosis Uncomplicated  Immune system disorder  Upper gastrointestinal cancers  Lung cancer  Tuberculosis  Infection Chronic (no other symptoms)  AIDS |
| Owen et al. (2023) | Diabetes  Congestive heart failure | | |
| Post et al. (2013) | Allergies  Arthritis  Asthma  Cancer  Chronic fatigue syndrome  Chronic menstrual irregularities (no male)  Diabetes  Fibromyalgia  Head injury (with loss of  consciousness)  Head injury (without loss of  consciousness)  Heart disease | Hypertension (high blood  pressure)  Hyperthyroidism  Hypoglycemia  Hypotension (low blood pressure)  Hypothyroidism  Irritable bowel syndrome  Kidney disease  Liver disease/Hepatitis  Migraine headache  Other  Seizure | |
| Public Health England (2018) | Asthma  Atrial fibrillation (AF)  Cancer  Coronary heart disease (CHD)  Chronic obstructive pulmonary disease (COPD)  Diabetes  Heart failure (HF)  Hypertension  Obesity  Stroke | | |
| Reilly et al. (2015) | Hypertension  Diabetes (type I and II)  Asthma  Hypothyroidism  Osteoarthritis  Chronic kidney disease (CKD), Learning disability  Coronary heart disease  Epilepsy  Chronic obstructive pulmonary disease (COPD)  Cancer  Stroke  Heart failure  Rheumatoid arthritis  Dementia  Psoriasis | | |
| Rodrigues et al. (2022) | Arthritis (either osteoarthritis or rheumatoid arthritis)  Asthma  Cancer  Congestive heart failure  Chronic obstructive pulmonary disease  Cardiovascular disease (CVD)  Dementia  Diabetes  HIV | Hypertension  Inflammatory bowel disease  Chronic kidney disease  Chronic liver disease  Osteoporosis  Stroke/transient ischemic attack  Urinary incontinence  Common mental disorder (either mood or anxiety disorder) | |
| Rojanaworarit et al. (2025) | Systemic diseases:  Hypertension  Diabetes mellitus  Cardiovascular disease  Thyroid disease  Anemia  Asthma  Epilepsy |  | |
| Smith et al. (2013a) | Coronary heart disease  Chronic kidney disease  Asthma (active)  Atrial fibrillation  Epilepsy  New cancer in the last 5 years  Thyrotoxicosis/thyroid disorders (includes hypothyroidism)  Diabetes  Parkinson’s disease  Multiple sclerosis  Stroke or transient ischaemic attack  Blindness and low vision  Glaucoma  Hearing loss  Hypertension  Heart failure | Peripheral vascular diseases  Chronic sinusitis  Bronchitis, emphysema and other chronic obstructive pulmonary diseases  Bronchiectasis  Crohn’s disease and ulcerative colitis  Diverticular disease of the intestine  Rheumatoid arthritis, other inflammatory polyarthropathies and systematic connective tissue disorders  Hyperplasia of prostate and prostate disorders  Psoriasis or eczema  Viral hepatitis  Irritable bowel syndrome  Cirrhosis/chronic liver disease/alcoholic liver disease  Migraine  Dyspepsia  Constipation  Pain | |
| Smith et al. (2013b) | Coronary heart disease  Chronic kidney disease  Asthma (active)  Atrial fibrillation  Epilepsy  New cancer in the last 5 years  Thyrotoxicosis/thyroid disorders (includes hypothyroidism)  Diabetes  Parkinson’s disease  Multiple sclerosis  Stroke or transient ischaemic attack  Blindness and low vision  Glaucoma  Hearing loss  Hypertension  Heart failure | Peripheral vascular diseases  Chronic sinusitis  Bronchitis, emphysema and other chronic obstructive pulmonary diseases  Bronchiectasis  Crohn’s disease and ulcerative colitis  Diverticular disease of the intestine  Rheumatoid arthritis, other inflammatory polyarthropathies and systematic connective tissue disorders  Hyperplasia of prostate and prostate disorders  Psoriasis or eczema  Viral hepatitis  Irritable bowel syndrome  Cirrhosis/chronic liver disease/alcoholic liver disease  Migraine  Dyspepsia  Constipation  Pain | |
| Stapp et al. (2020) | Arteriosclerosis  Hypertension  Cirrhosis  Other liver disease  Angina  Tachycardia  Myocardial infarction  Other heart disease  Stomach ulcer  Gastritis  Arthritis | | |
| Stubbs et al. (2016) | Arthritis  Angina pectoris  Asthma  Diabetes  Chronic back pain  Visual impairment  Hearing problems  Edentulism  Tuberculosis | | |
| Teh et al. (2021) | Respiratory conditions (asthma, emphysema)  Diabetes or hyperglycemia  Hypertension  Hyperlipidemia  Chronic pain (arthritis, rheumatism, migraine, back problems)  Cancer  Neurological disorders (epilepsy, Parkinson’s disease)  Cardiovascular diseases (stroke, heart attack, angina, or other heart diseases)  Ulcer and chronic inflamed bowel (stomach ulcer, enteritis, colitis). | | |
| Thabet et al. (2019) | Diabetes  Overweight/obesity  Dyslipidemia  Heart diseases  Metabolic syndrome  Bone pathologies  Respiratory pathologies  Sexual dysfunction | | |
| **Study** | **Physical health conditions studied** | | |
| Bouza et al. (2010) | Infectious diseases  Neoplasms  Endocrine diseases  Hematological diseases  Neurological diseases  Diseases of the circulatory system  Respiratory diseases  Diseases of the digestive system  Diseases of the genitourinary tract  Complications of pregnancy, childbirth, and the puerperium  diseases of the skin and subcutaneous tissue  Diseases of the musculoskeletal system and connective tissue  Injury and poisoning  Drug, alcohol and tobacco abuse/dependency | | |
| Charlson et al. (2021) | Non-communicable diseases:  Diabetes  Cardiovascular disease  Respiratory disease  Renal disease  Neurological disease  Communicable diseases (sexually transmitted infections [STIs])  Injuries (excluding self-harm) | | |
| de Freitas et al. (2022) | Asthma  Bronchitis  Diabetes  Hypertension  Low blood pressure  Overweight or obesity  Rheumatoid arthritis | | |
| Dixon et al. (1999) | High blood pressure  Diabetes  Sexually transmitted diseases (STDs)  Cancer  Breathing problems  Heart problems  Bowel problems  Hearing problems  Eyesight problems  Teeth problems  Skin problems  Seizures | | |
| Filipcic et al. (2019) | Asthma (allergic asthma included)  Chronic bronchitis, chronic obstructive pulmonary disease, and emphysema  Myocardial infarction (heart attack) or chronic consequences of myocardial infarction  Coronary heart disease or angina pectoris  High blood pressure (hypertension)  Cerebrovascular insult (cerebral haemorrhage, cerebral thrombosis) or chronic consequences of stroke  Arthrosis (arthritis excluded)  Low back disorder or other chronic back defects  Neck disorder or other chronic neck defects  Diabetes mellitus  Allergy (rhinitis, hay fever, eye inflammation, dermatitis, food allergy, or other allergies [allergic asthma excluded]  Liver cirrhosis  Urinary incontinence, problems in controlling the bladder  Kidney disease  Obesity | | |
| Gabilondo et al. (2017) | Parkinson  Dementia  Viral hepatitis  Chronic constipation  Human immunodeficiency virus Diabetes  Emphysema, chronic bronchitis, chronic obstructive pulmonary disease Chronic liver or pancreatic disease  Psoriasis or eczema  Hypothyroidism  Dyspepsia  Epilepsy (currently treated)  Heart failure  Disorders of the immune system  Paralysis or muscular dystrophy  Blindness & low vision  Cerebrovascular disease  Hematologic chronic disorders  Low back pain  Irritable bowel syndrome  Deafness  Chronic kidney disease  Glaucoma | Transplant status  Chronic heart disease, others  Chromosomal anomalies or inherited metabolic disorders  Bronchiectasis  Inflammatory bowel disease  Asthma (currently treated)  Multiple sclerosis  Hypertension  Ischemic heart disease  Cancer  Prostatic hypertrophy  Peripheral vascular disease  Peripheral neuropathy  Chronic sinusitis  Diverticular disease of intestine  Gout  Atrial fibrillation  Degenerative joint disease  Rheumatoid arthritis and autoimmune and connective tissue diseases Osteoporosis  Migraine | |
| García-Goñi et al. (2021) | Asthma  Chronic kidney disease  Diabetes  Chronic obstructive pulmonary disease  Heart failure  HIV  Migraine  Multiple sclerosis | | |
| Godin et al. (2023) | Neurological disorders (headache/migraine, multiple sclerosis, epilepsy, meningitis, stroke, head injury)  Cardiovascular disorders (hypertension, coronary disease, myocardial infarction, cardiac dysrhythmia)  Endocrine and metabolic disorders (diabetes, thyroid disorders, hypercholesterolemia, hypertriglyceridemia)  Urinary tract disorders (nephropathy, acute retention of urine)  Skin disorders (psoriasis, eczema, toxidermia/rash, acne)  Liver and digestive disorders (cirrhosis, hepatitis, peptic ulcer, inflammatory chronic intestinal diseases)  Allergic and auto-immune-inflammatory disorders (asthma, allergy, systemic lupus erythematosus, rheumatoid polyarthritis)  Cancer and chronic infectious disorders (HIV, chronic viral hepatitis),  Hypertension  Hyperglycemia  Hypercholesterolemia  Hypertriglyceridemia | | |
| Hosang et al. (2017) | Heart problems (i.e. stroke, angina and heart attack)  Asthma  Diabetes (I and II)  Arthritis (i.e. osteoarthritis, rheumatoid arthritis and other types of arthritis)  Hypertension  Epilepsy or convulsions  Osteoporosis  Multiple sclerosis  Emphysema or chronic bronchitis  Post herpetic neuralgia | | |
| Hosang et al. (2018) | Heart problems (stroke, angina and heart attack)  Asthma  Diabetes (type 1 and type 2)  Arthritis (osteoarthritis and rheumatoid arthritis)  Hypertension  Epilepsy or convulsions  Osteoporosis  Multiple sclerosis | | |
| Jahrami et al. (2017) | Diabetes type 2  Hypertension  Cardiovascular problems (angina, myocardial infarction, stroke, heart  failure, cardiomyopathy, heart arrhythmia, carditis, and venous  thrombosis)  Musculoskeletal disorders (osteoarthritis, gout,  rheumatoid arthritis, fibromyalgia, plantar fasciitis, heel spurs, and tendonitis) | | |
| Lasebikan & Azegbeobor (2017) | Nutritional anaemia  Hypertension  Ischaemic heart disease  Bronchial asthma  Chronic dermatitis  Diabetes mellitus  Thyrotoxicosis  Hypothyroidism  Chronic pain  Chronic headache  Peptic ulcer disease  Osteoarthritis haemorrhoids Malignancy  Common chronic infections such as tuberculosis, schistosomiasis, dracontiasis, malaria, hepatitis | | |
| Launders et al. (2022) | Asthma  COPD  Cardiac arrhythmia  Congestive heart failure  Myocardial infarction  Cerebrovascular disease  Neurological disorders (including epilepsy, multiple sclerosis, Parkinson disease, and seizures, but excluding cerebrovascular disease and dementia)  Cancer  Diabetes (type 1 or 2)  Hypothyroidism  Liver disease  Renal disease | Peptic ulcers  Rheumatic and collagen disease  Paresis or paralysis  HIV/AIDS  Hypertension  Peripheral vascular disease  Pulmonary circulation disorders  Valvular disease  Deficiency anaemia  Blood loss anaemia  Coagulopathy  Fluid or electrolyte disorders | |
| Mirza et al. (2021) | Neoplastic disorders  Haematological disorders  Endocrine disorders  Neurological disorders  Eye and adnexal disorders  Circulatory disorders  Respiratory disorders  Digestive disorders  Dermatological disorder  Musculoskeletal disorders  Genitourinary disorders | | |
| Owen et al. (2023) | Diabetes  Congestive heart failure | | |
| Post et al. (2013) | Allergies  Arthritis  Asthma  Cancer  Chronic fatigue syndrome  Chronic menstrual irregularities (no male)  Diabetes  Fibromyalgia  Head injury (with loss of  consciousness)  Head injury (without loss of  consciousness)  Heart disease | Hypertension (high blood  pressure)  Hyperthyroidism  Hypoglycemia  Hypotension (low blood pressure)  Hypothyroidism  Irritable bowel syndrome  Kidney disease  Liver disease/Hepatitis  Migraine headache  Other  Seizure | |
| Public Health England (2018) | Asthma  Atrial fibrillation (AF)  Cancer  Coronary heart disease (CHD)  Chronic obstructive pulmonary disease (COPD)  Diabetes  Heart failure (HF)  Hypertension  Obesity  Stroke | | |
| Reilly et al. (2015) | Hypertension  Diabetes (type I and II)  Asthma  Hypothyroidism  Osteoarthritis  Chronic kidney disease (CKD), Learning disability  Coronary heart disease  Epilepsy  Chronic obstructive pulmonary disease (COPD)  Cancer  Stroke  Heart failure  Rheumatoid arthritis  Dementia  Psoriasis | | |
| Rodrigues et al. (2022) | Arthritis (either osteoarthritis or rheumatoid arthritis)  Asthma  Cancer  Congestive heart failure  Chronic obstructive pulmonary disease  Cardiovascular disease (CVD)  Dementia  Diabetes  HIV | Hypertension  Inflammatory bowel disease  Chronic kidney disease  Chronic liver disease  Osteoporosis  Stroke/transient ischemic attack  Urinary incontinence  Common mental disorder (either mood or anxiety disorder) | |
| Smith et al. (2013a) | Coronary heart disease  Chronic kidney disease  Asthma (active)  Atrial fibrillation  Epilepsy  New cancer in the last 5 years  Thyrotoxicosis/thyroid disorders (includes hypothyroidism)  Diabetes  Parkinson’s disease  Multiple sclerosis  Stroke or transient ischaemic attack  Blindness and low vision  Glaucoma  Hearing loss  Hypertension  Heart failure | Peripheral vascular diseases  Chronic sinusitis  Bronchitis, emphysema and other chronic obstructive pulmonary diseases  Bronchiectasis  Crohn’s disease and ulcerative colitis  Diverticular disease of the intestine  Rheumatoid arthritis, other inflammatory polyarthropathies and systematic connective tissue disorders  Hyperplasia of prostate and prostate disorders  Psoriasis or eczema  Viral hepatitis  Irritable bowel syndrome  Cirrhosis/chronic liver disease/alcoholic liver disease  Migraine  Dyspepsia  Constipation  Pain | |
| Smith et al. (2013b) | Coronary heart disease  Chronic kidney disease  Asthma (active)  Atrial fibrillation  Epilepsy  New cancer in the last 5 years  Thyrotoxicosis/thyroid disorders (includes hypothyroidism)  Diabetes  Parkinson’s disease  Multiple sclerosis  Stroke or transient ischaemic attack  Blindness and low vision  Glaucoma  Hearing loss  Hypertension  Heart failure | Peripheral vascular diseases  Chronic sinusitis  Bronchitis, emphysema and other chronic obstructive pulmonary diseases  Bronchiectasis  Crohn’s disease and ulcerative colitis  Diverticular disease of the intestine  Rheumatoid arthritis, other inflammatory polyarthropathies and systematic connective tissue disorders  Hyperplasia of prostate and prostate disorders  Psoriasis or eczema  Viral hepatitis  Irritable bowel syndrome  Cirrhosis/chronic liver disease/alcoholic liver disease  Migraine  Dyspepsia  Constipation  Pain | |
| Stapp et al. (2020) | Arteriosclerosis  Hypertension  Cirrhosis  Other liver disease  Angina  Tachycardia  Myocardial infarction  Other heart disease  Stomach ulcer  Gastritis  Arthritis | | |
| Stubbs et al. (2016) | Arthritis  Angina pectoris  Asthma  Diabetes  Chronic back pain  Visual impairment  Hearing problems  Edentulism  Tuberculosis | | |
| Teh et al. (2021) | Respiratory conditions (asthma, emphysema)  Diabetes or hyperglycemia  Hypertension  Hyperlipidemia  Chronic pain (arthritis, rheumatism, migraine, back problems)  Cancer  Neurological disorders (epilepsy, Parkinson’s disease)  Cardiovascular diseases (stroke, heart attack, angina, or other heart diseases)  Ulcer and chronic inflamed bowel (stomach ulcer, enteritis, colitis). | | |
| Thabet et al. (2019) | Diabetes  Overweight/obesity  Dyslipidemia  Heart diseases  Metabolic syndrome  Bone pathologies  Respiratory pathologies  Sexual dysfunction | | |

Supplementary Table 8. Search strategy

| **Concept** | **PsychINFO** | **Embase** | **PubMed** |
| --- | --- | --- | --- |
| **Severe mental illness** | 1. (psychosis or psychotic or psychotic disorder* or schizophreni* or sever* mental ill* or sever* mental disorder* or SMI or schizoaffective disorder* or schizo-affective disorder*).ti,ab. | 1. (psychosis or psychotic or psychotic disorder* or schizophreni* or sever* mental ill* or sever* mental disorder* or SMI or schizoaffective disorder* or schizo-affective disorder*).ti,ab,kw. | 1. psychosis[Title/Abstract] or psychotic[Title/Abstract] or "psychotic disorder*"[Title/Abstract] or schizophreni*[Title/Abstract] or "severe mental ill*"[Title/Abstract] or "severe mental disorder*"[Title/Abstract] or SMI[Title/Abstract] or "schizoaffective disorder*"[Title/Abstract] or "schizo-affective disorder*"[Title/Abstract] OR "Schizophrenia"[Mesh] OR "Schizophrenia Spectrum and Other Psychotic Disorders"[Mesh] OR "Schizophrenia, Paranoid"[Mesh] |
| **Multimorbidity** | 2. (multimorbidit* or multiple chronic condition* or multiple condition* or multiple diagnos* or multiple disease* or multiple health problem* or multiple illness* or multiple patholog* or multidisease* or multi-disease* or multi-morbidit* or multipatholog* or multi-patholog* or pluripatholog* or polypatholog* or poly-patholog* or multiple long term condition* or multiple long-term condition* or multiple long term illness* or multiple long-term illness* or multiple chronic illness* or multiple health condition* or multiple physical health condition* or multiple physical health illness* or multiple physical health problem*).ti,ab. | 2. (multimorbidit* or multiple chronic condition* or multiple condition* or multiple diagnos* or multiple disease* or multiple health problem* or multiple illness* or multiple patholog* or multidisease* or multi-disease* or multi-morbidit* or multipatholog* or multi-patholog* or pluripatholog* or polypatholog* or poly-patholog* or multiple long term condition* or multiple long-term condition* or multiple long term illness* or multiple long-term illness* or multiple chronic illness* or multiple health condition* or multiple physical health condition* or multiple physical health illness* or multiple physical health problem*).ti,ab,kw. | 2. multimorbidit*[Title/Abstract] or "multiple chronic condition*"[Title/Abstract] or "multiple condition*"[Title/Abstract] or "multiple diagnos*"[Title/Abstract] or "multiple disease*"[Title/Abstract] or "multiple health problem*"[Title/Abstract] or "multiple illness*"[Title/Abstract] or "multiple patholog*"[Title/Abstract] or multidisease*[Title/Abstract] or multi-disease*[Title/Abstract] or multi-morbidit*[Title/Abstract] or multipatholog*[Title/Abstract] or multi-patholog*[Title/Abstract] or pluripatholog*[Title/Abstract] or polypatholog*[Title/Abstract] or poly-patholog*[Title/Abstract] or "multiple long term condition*"[Title/Abstract] or "multiple long-term condition*"[Title/Abstract] or "multiple long term illness*"[Title/Abstract] or "multiple long-term illness*"[Title/Abstract] or "multiple chronic illness*"[Title/Abstract] or "multiple health condition*"[Title/Abstract] or "multiple physical health condition*"[Title/Abstract] or "multiple physical health illness*"[Title/Abstract] or "multiple physical health problem*"[Title/Abstract] or "Multimorbidity"[Mesh] OR "Multiple Chronic Conditions"[Mesh] |
| **Comorbidity** | 3. (comorbidity or comorbidities or co-occurrence or comorbid).ti,ab. | 3. (comorbidity or comorbidities or co-occurrence or comorbid).ti,ab,kw. | 3. comorbidity[Title/Abstract] or comorbidities[Title/Abstract] or co-occurrence[Title/Abstract] or comorbid[Title/Abstract] |
| **Risk factors** | 4. (risk factor* or correlate* or antecedent* or causal* or causal factor* or protective factor* or determinant* or predict* or predictive factor*).ti,ab. | 4. (risk factor* or correlate* or antecedent* or causal* or causal factor* or protective factor* or determinant* or predict* or predictive factor*).ti,ab,kw. | 4. risk factor*[Title/Abstract] or correlate*[Title/Abstract] or antecedent*[Title/Abstract] or causal*[Title/Abstract] or causal factor*[Title/Abstract] or protective factor*[Title/Abstract] or determinant*[Title/Abstract] or predict*[Title/Abstract] or predictive factor*[Title/Abstract] |
| **Linking concepts** | 5. 1 and 2 and 4 6. 1 and 3 and 4 7. 5 or 6 | 5. 1 and 2 and 4 6. 1 and 3 and 4 7. 5 or 6 | 5. 1 and 2 and 4 6. 1 and 3 and 4 7. 5 or 6 |
| **Limits** | English language only | English language only | English language only |

Supplementary Table 9. PRISMA checklist

| **Section and Topic** | **Item #** | **Checklist item** | **Location where item is reported** |
| --- | --- | --- | --- |
| **TITLE** | | |  |
| Title | 1 | Identify the report as a systematic review. | Page 1 |
| **ABSTRACT** | | |  |
| Abstract | 2 | See the PRISMA 2020 for Abstracts checklist. | Page 2 |
| **INTRODUCTION** | | |  |
| Rationale | 3 | Describe the rationale for the review in the context of existing knowledge. | Pages 3-4 |
| Objectives | 4 | Provide an explicit statement of the objective(s) or question(s) the review addresses. | Page 4 |
| **METHODS** | | |  |
| Eligibility criteria | 5 | Specify the inclusion and exclusion criteria for the review and how studies were grouped for the syntheses. | Page 6; Supplementary Table 1 |
| Information sources | 6 | Specify all databases, registers, websites, organisations, reference lists and other sources searched or consulted to identify studies. Specify the date when each source was last searched or consulted. | Page 5 |
| Search strategy | 7 | Present the full search strategies for all databases, registers and websites, including any filters and limits used. | Supplementary Table 8 |
| Selection process | 8 | Specify the methods used to decide whether a study met the inclusion criteria of the review, including how many reviewers screened each record and each report retrieved, whether they worked independently, and if applicable, details of automation tools used in the process. | Page 6 |
| Data collection process | 9 | Specify the methods used to collect data from reports, including how many reviewers collected data from each report, whether they worked independently, any processes for obtaining or confirming data from study investigators, and if applicable, details of automation tools used in the process. | Page 6-7 |
| Data items | 10a | List and define all outcomes for which data were sought. Specify whether all results that were compatible with each outcome domain in each study were sought (e.g. for all measures, time points, analyses), and if not, the methods used to decide which results to collect. | Page 6-7 |
|  | 10b | List and define all other variables for which data were sought (e.g. participant and intervention characteristics, funding sources). Describe any assumptions made about any missing or unclear information. | Page 6-7 |
| Study risk of bias assessment | 11 | Specify the methods used to assess risk of bias in the included studies, including details of the tool(s) used, how many reviewers assessed each study and whether they worked independently, and if applicable, details of automation tools used in the process. | Page 7 |
| Effect measures | 12 | Specify for each outcome the effect measure(s) (e.g. risk ratio, mean difference) used in the synthesis or presentation of results. | Page 7 |
| Synthesis methods | 13a | Describe the processes used to decide which studies were eligible for each synthesis (e.g. tabulating the study intervention characteristics and comparing against the planned groups for each synthesis (item #5)). | Page 7 |
|  | 13b | Describe any methods required to prepare the data for presentation or synthesis, such as handling of missing summary statistics, or data conversions. | Page 7 |
|  | 13c | Describe any methods used to tabulate or visually display results of individual studies and syntheses. | Page 7 |
|  | 13d | Describe any methods used to synthesize results and provide a rationale for the choice(s). If meta-analysis was performed, describe the model(s), method(s) to identify the presence and extent of statistical heterogeneity, and software package(s) used. | Page 7 |
|  | 13e | Describe any methods used to explore possible causes of heterogeneity among study results (e.g. subgroup analysis, meta-regression). | N/A |
|  | 13f | Describe any sensitivity analyses conducted to assess robustness of the synthesized results. | N/A |
| Reporting bias assessment | 14 | Describe any methods used to assess risk of bias due to missing results in a synthesis (arising from reporting biases). | Page 7 |
| Certainty assessment | 15 | Describe any methods used to assess certainty (or confidence) in the body of evidence for an outcome. | Page 7 |
| **RESULTS** | | |  |
| Study selection | 16a | Describe the results of the search and selection process, from the number of records identified in the search to the number of studies included in the review, ideally using a flow diagram. | Page 8; Figure 1 |
|  | 16b | Cite studies that might appear to meet the inclusion criteria, but which were excluded, and explain why they were excluded. | Figure 1 |
| Study characteristics | 17 | Cite each included study and present its characteristics. | Pages 8-10; Table 1 |
| Risk of bias in studies | 18 | Present assessments of risk of bias for each included study. | Page 8; Supplementary Table 6 |
| Results of individual studies | 19 | For all outcomes, present, for each study: (a) summary statistics for each group (where appropriate) and (b) an effect estimate and its precision (e.g. confidence/credible interval), ideally using structured tables or plots. | Supplementary Table 2 and 3 |
| Results of syntheses | 20a | For each synthesis, briefly summarise the characteristics and risk of bias among contributing studies. | Pages 8-20 |
|  | 20b | Present results of all statistical syntheses conducted. If meta-analysis was done, present for each the summary estimate and its precision (e.g. confidence/credible interval) and measures of statistical heterogeneity. If comparing groups, describe the direction of the effect. | Pages 8-20 |
|  | 20c | Present results of all investigations of possible causes of heterogeneity among study results. | Pages 8-20 |
|  | 20d | Present results of all sensitivity analyses conducted to assess the robustness of the synthesized results. | N/A |
| Reporting biases | 21 | Present assessments of risk of bias due to missing results (arising from reporting biases) for each synthesis assessed. | Supplementary Table 10 |
| Certainty of evidence | 22 | Present assessments of certainty (or confidence) in the body of evidence for each outcome assessed. | N/A |
| **DISCUSSION** | | |  |
| Discussion | 23a | Provide a general interpretation of the results in the context of other evidence. | Pages 21-26 |
|  | 23b | Discuss any limitations of the evidence included in the review. | Pages 24-25 |
|  | 23c | Discuss any limitations of the review processes used. | Pages 25-26 |
|  | 23d | Discuss implications of the results for practice, policy, and future research. | Pages 23-24 |
| **OTHER INFORMATION** | | |  |
| Registration and protocol | 24a | Provide registration information for the review, including register name and registration number, or state that the review was not registered. | Page 5 |
|  | 24b | Indicate where the review protocol can be accessed, or state that a protocol was not prepared. | Page 5 |
|  | 24c | Describe and explain any amendments to information provided at registration or in the protocol. | N/A |
| Support | 25 | Describe sources of financial or non-financial support for the review, and the role of the funders or sponsors in the review. | Page 1 |
| Competing interests | 26 | Declare any competing interests of review authors. | Page 1 |
| Availability of data, code and other materials | 27 | Report which of the following are publicly available and where they can be found: template data collection forms; data extracted from included studies; data used for all analyses; analytic code; any other materials used in the review. | N/A |

Supplementary Table 10. Completed ROBIS tool


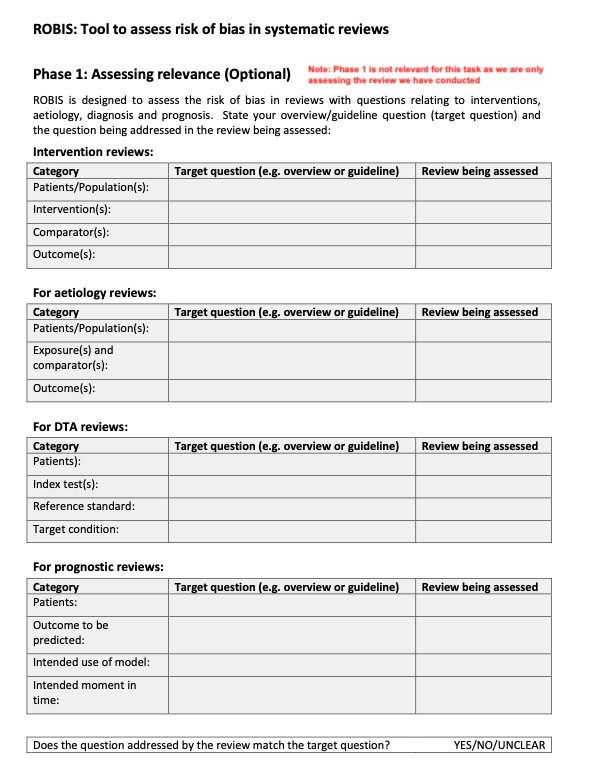


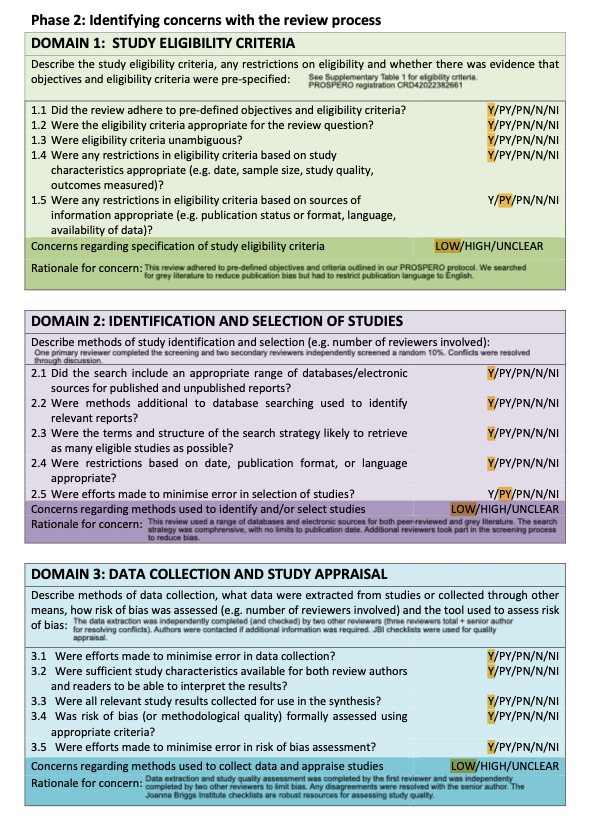


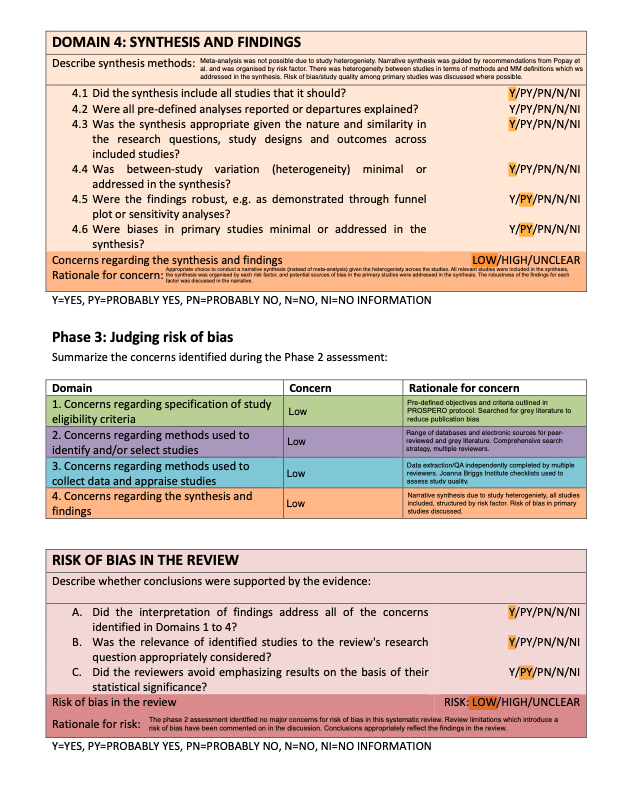

Supplement: R_and_R_Supplementary_material_sbaf128 [file r_and_r_supplementary_material_sbaf128.doc]
